# TAB182 aggravates progression of esophageal squamous cell carcinoma by enhancing β-catenin nuclear translocation through FHL2 dependent manner

**DOI:** 10.1038/s41419-022-05334-2

**Published:** 2022-10-26

**Authors:** Aidi Gao, Zhenzi Su, Zengfu Shang, Chao He, Dongliu Miao, Xiaoqing Li, Shitao Zou, Weiqun Ding, Yue Zhou, Ming Sun, Jundong Zhou

**Affiliations:** 1 Suzhou Cancer Center Core Laboratory, The Affiliated Suzhou Hospital of Nanjing Medical University, Suzhou, Jiangsu P.R. China; 2 The Affiliated Suqian Hospital of Xuzhou Medical University and Nanjing Drum Tower Hospital Group Suqian Hospital, Suqian, Jiangsu P.R. China; 3 School of Radiation Medicine and Protection, Medical College of Soochow University, Suzhou, China; 4 Department of Pathology, University of Oklahoma Health Science Center, Oklahoma City, OK USA; 5 Department of Thoracic Surgery, First Affiliated Hospital of Nanjing Medical University, Nanjing, China; 6 Suzhou Cancer Center Core Laboratory, The Affiliated Suzhou Hospital of Nanjing Medical University, Suzhou Municipal Hospital, Gusu School, Baita west road #16, 215001 Suzhou, China

**Keywords:** Oncogenes, Cell growth

## Abstract

TAB182 (also named TNKS1BP1), a binding protein of tankyrase 1, has been found to participate in DNA repair. Our previous study has revealed the involvement of TAB182 in the radioresistance of esophageal squamous cell carcinoma (ESCC) cells. However, whether TAB182 contributes to the ESCC tumorigenesis and progression remains unclear. In this study, we found that highly expressed TAB182 is closely associated with a poor prognosis of patients with ESCC. TAB182 silencing reduced ESCC cell proliferation and invasion in vitro, tumorigenicity and metastasis in vivo. RNA-seq and IP-MS analysis revealed that TAB182 could affect the β-catenin signaling pathway via interacting with β-catenin. Furthermore, TAB182 prevented β-catenin to be phosphorylated by GSK3β and recruited four and a half of LIM-only protein 2 (FHL2), which thereby promoted β-catenin nucleus translocation to result in activation of the downstream targets transcription in ESCC cells. Our findings demonstrate that TAB182 enhances tumorigenesis of esophageal cancer by promoting the activation of the β-catenin signaling pathway, which provides new insights into the molecular mechanisms by which TAB182 accelerates progression of ESCC.

## Introduction

Esophageal cancer (EC) is the sixth most prevalently diagnosed malignancy in the world with estimated 450,000 deaths every year. Strikingly, China accounts for half of the global morbidity as well as mortality of EC [1, 2]. Esophageal squamous cell carcinoma (ESCC), as well as esophageal adenocarcinoma (EAC), is the primary histological types of EC. In China, ESCC is the most predominant subtype with high incidences, accounting for 90% of newly diagnosed EC cases [3, 4]. To date, the prognosis among patients suffering from ESCC is still poor, with a 5-year overall survival (OS) rate of less than 25% [5]. A better comprehension of the underlying mechanism of ESCC pathogenesis can facilitate the developing of novel therapeutic and diagnostic strategies to improve ESCC outcome.

TAB182, also referred to as TNKS1BP1, was first discovered as a tankyrase 1-binding protein that acts as an receptor of poly (ADP-ribosyl) action by tankyrase 1 [6]. Recently, Zhong et al. reported that TAB182 is up-regulated in lung adenocarcinoma and affects lung adenocarcinoma cells’ sensitivity to DNA damage regent through regulating the homologous recombination pathway of DNA double-strand breaks (DSB) [7]. Moreover, Zhou and colleagues found that TAB182 could be induced by ionizing radiation (IR), and TAB182 is required for the efficient repair of IR-mediated DSB through enhancing DNA-PKcs’ interacting with PARP-1 [8]. Our previous study showed that TAB182 heightens the radioresistance of ESCC cells by mediating the G2-M checkpoint via its interaction with FHL2 [9]. Although recent studies have revealed the crucial roles of TAB182 in DNA damage response, whether and how TAB182 associates with ESCC tumorigenesis and progression remain unclear.

FHL2 is a multifunctional scaffolding protein that belongs to the four-and-a-half LIM domains protein family. FHL2 has been found to interact with multiple types of proteins due to its unique structure that consists of several LIM motifs, including IER3 and MDM2 [10], EGFR, and EGFRvIII [11]. Additionally, it is reported that the zinc-finger motif in the LIM domain confers FHL2 with transcriptional repressor or co-activator function [12, 13]. As a result, FHL2 is involved in the modulation of varied cellular processes, including gene expression, cell proliferation and motility [14]. Interestingly, accumulating evidence has demonstrated the critical roles of FHL2 in tumorigenesis and the progression of carcinoma in a context-dependent manner. For instance, it exerts oncogenic function in ovarian cancer [15], cervical cancer [10], and gastrointestinal cancer [16], but exhibits a tumor-suppressive function in rhabdomyosarcoma [17] and hepatocellular carcinoma [18], which indicates that FHL2 interacts with different partners that might lead to varying downstream effects.

In this study, we explored whether TAB182 influences the onset as well as the progression of ESCC. We found that TAB182 is highly expressed in ESCC, and down-regulation of TAB182 impaired ESCC cell proliferation, invasion, and cancer stem-like characteristics. Mechanistic studies reveal that TAB182 physically binds with β-catenin and prevents it from being phosphorylated by GSK3β, and further recruits FHL2 to facilitate β-catenin nucleus translocation and downstream target genes transcription. These findings demonstrate that TAB182 is an oncogenic regulator that accelerate the development and progression of ESCC via regulating the β-catenin signaling pathway.

## Materials and methods

### ESCC cell lines

Human ESCC cell lines TE-10 and KYSE-150 were purchased from ATCC (Manassas, Virginia, USA). TE-10 and KYSE-150 were cultured in RPMI-1640 (Hyclone, USA, Cat# SH30B09.01) with 10% fetal bovine serum (FBS, Biological Industries, Israel, REF# 04-001-1ACS), 100 U/mL penicillin G, and 100 μg/mL streptomycin (Hyclone, USA, Cat# SV30010) with sustained parameters of the humidified atmosphere of 5% CO_2_ at 37 °C. For the establishment of TAB182-overexpressed or -silenced cells, human TAB182 cDNA and targeted shRNA were cloned into lentivirus-based vectors. The expression of TAB182 in the virus-infected human EC cell lines TE-10 and KYSE-150 was confirmed by western blotting.

### Cell transfection

TE-10 and KYSE-150 cells were seeded in six-well plates one day before transfection and transiently transfected with siRNAs using RNAiMAX (Invitrogen, USA, Cat# 13778150). 48 h after transfection, cells were harvested and analyzed by qRT-PCR and western blot. HEK293T cells were transfected with TAB182-WT, TAB182Δ1-2 and FHL2Δ1-4 plasmids using Lipofectamine3000 Transfection Kit (Invitrogen, USA, Cat# L3000015). Cells transfected with PCDNA3.0 vector only served as control. The ESCC cell lines were infected with the lentiviruses that contain TAB182 et al. genes shRNA or CDS sequence, and stable knockdown or over-expression cell lines were produced by selection with 1 μg/ml puromycin (Beyotime, China, Cat# ST551).

### Quantitative real-time PCR (qRT-PCR)

Trizol Reagent was utilized in the extraction of total RNA in ESCC cells following the manufacturer’s specifications. The cDNA was produced by reverse transcription utilizing RevertAidTM First-Strand cDNA Synthesis Kit (Thermo Scientific, USA, Cat# K1621) and oligo (dT) in a 20-µL reaction mixture containing 1 µg of total RNA. qRT-PCR was conducted utilizing QuantiNova SYBR Green qPCR kit (QIAGEN, Germany, Cat# 208054) on an ABI Prism 7500 real-time PCR system, according to the manufacturer’s specifications. Threshold cycle (Ct) values of TAB182 and other different mRNAs were equilibrated to that of β-actin, which was utilized as an internal control. The relative expression was computed utilizing the 2^-ΔΔCt^ approach. The primers used in this study were listed in the Table S1.

### Western blotting

Cellular proteins from TE-10 and KYSE-150 cells were obtained using the M-PER Mammalian Protein Extraction Kit (Thermo Scientific, USA, Cat# 78501) following the manufacturer’s protocols. Total protein or nuclear protein was extracted using the Nuclear (Nucleic Acid-Free) extraction kit (Abcam, USA, Cat# ab113477). The same amount of protein was loaded in every lane and resolved by SDS-PAGE utilizing a Tris-glycine running buffer. The separated proteins were placed onto nitrocellulose membranes. The membranes were blocked with 5% non-fat milk before undergoing incubation with primary antibodies at 4 °C overnight, followed by incubation with the HRP-coupled secondary antibody for 1 h at room temperature. The visualization of the blots was achieved with the aid of enhanced chemiluminescence detection reagents (NCM biotech, China, Cat# P10100). The blots were stripped and re-probed with the HRP-labeled anti-β-Tubulin antibody. Antibodies to TAB182, FHL2, β-catenin, and β-Tubulin were procured from Cell Signaling Technology (Boston, MA, USA).

### Cell invasion assay

The TE-10 as well as KYSE-150 cells mentioned above were seeded into the upper Transwell chamber coated with Corning Matrigel matrix (Sigma, USA, REF# 356234) in a serum-free RPMI-1640 medium and incubated at 37 °C, with 5% CO_2_ for 24 h. The invaded cells from the upper Transwell chamber to the lower chamber were stained using the Wright–Giemsa solution (Nanjing JianCheng Technology, China, Cat# D010) before imaging them. Cells in six randomly chosen fields of the lower chamber were then counted.

### CCK8 and colony formation assay

CCK8 reagent was used to evaluate the proliferation ability of ESCC cells. In brief, 1000 TE-10 or KYSE-150 cells were seeded into each well of 96-well plate. Then, 10 μL of CCK8 regent (Vazyme, China, Cat# A311-01) was added onto each well, followed with incubation for 2 h at 37 °C. Then, the absorbance value of the cells at 450 nm was examined. For the colony formation assay, the 35-mm tissue culture plate was coated with 0.5% agarose supplemented with RPMI-1640 complete medium. Upon solidification of the bottom layer, 1 × 10^3^ TE-10 or KYSE-150 cells in 1.6 mL of the complete medium were mixed with 0.15 mL of 4% low-melting agarose and transferred to the plates to allow it to solidify. Subsequently, the dishes were subjected to incubation at 37 °C in an atmosphere with 5% CO_2_ for 14 days. The Zoom-Stereo Microscope SZX16 (OLYMPUS, Tokyo, Japan) was utilized in counting as well as visualizing the colonies.

### Tumor sphere formation assay

TE-10 and KYSE-150 cells were cultured to *logarithmic* growth stage utilizing a complete medium or a serum-free F12 medium supplemented with 10 ng/ml EGF (Thermo Scientific, USA, Cat# PHG0311), 10 ng/ml bFGF (Thermo Scientific, USA, Cat# PHG0368), and 1×B27 (Thermo Scientific, USA, Cat# 12587-010) under a humidified atmosphere of 5% CO_2_ at 37 °C. Afterward, Intelligent Bio-image navigation FSX100 (Olympus Optical Co., Ltd, Tokyo, Japan) was used to image the cells. Tumor spheres denoted spheres that had greater than 50 mammary cells.

### ESCC cell xenograft and metastasis model in mice

6-week-old female nude mice with a weight range of 18–22 g were subcutaneously administered with human EC cells through injection (1 × 10^7^ cells per mice). In this experiment, mice were randomly grouped (*n* = 6). After 18 days, the tumors in mice were excised and weighed. Then, the body weight of mice and tumors were measured and monitored each alternate day. The tumor volume was established utilizing this formula: length × width^2^ × 0.5. At the end of 40 days, the mice were killed to allow the harvesting of the tumors. For the metastasis mouse model, 1 × 10^7^ KYSE-150 cells in 150 µL PBS were administered into tail veins of 6-week-old female nude mice through injection. 45 days following injection, lung tissues were collected for detection of cancer cell metastasis. The metastatic nodules were determined by pathological HE staining. This research was subjected to approval by the Research Ethics Committee of The Affiliated Suzhou Hospital of Nanjing Medical University.

### Microarray and gene expression profile analysis

The gene expression levels in KYSE-150-NC and KYSE-150-TAB182 KD (knockdown) cells were assessed using the Illumina Genome AnalyzerIIx (NovelBio Bio-Pharm Technology Co, Ltd, Shanghai, China; with 48,000 transcripts and 32,375 human genes, including cDNA controls). The genes upregulated and downregulated by approximately 1.5-folds (p value<0.05) in the KYSE-150-TAB182 KD cells compared with the expression levels in KYSE-150-NC control cells were analyzed using the Affy package in R language (v3.4.4).

### Immunoprecipitation (IP)

The ESCC cells underwent lysis utilizing lysis buffer from the M-PER Mammalian Protein Extraction Kit (Thermo Scientific, USA, Cat# 78501). Cell lysates underwent incubation with TAB182, FHL2, or β-catenin antibodies at 4 °C overnight before again incubating them with the protein G-beads (Abcam, USA, Cat# ab193262) pre-blocked using 5% BSA (Multi Sciences, China, Cat# A3828-100). The beads were then washed four times using 0.1% Triton-PBS buffer, suspended in 0.1 mL of 1× SDS sample buffer and 1 mM DTT (Solarbio, Cat# 3482-12-3), and centrifuged at the rate of 13,000× *g* for 10 min. The eluates from the IP beads were used in Western blotting to confirm the interaction between TAB182 and the proteins mentioned above.

### Immunofluorescence (IF) staining

The cells were seeded on the slides and cultured at 37 °C, with 5% CO_2_ for 24 h. Additionally, the cells were exposed to 4% paraformaldehyde (Biosharp, China, REF# BL539A) for 10 min at room temperature in order to fix them, accompanied by incubation with 0.1% Triton X-100 (Solarbio, China, Cat# T8200) for 15 min to facilitate the permeation of the cells. The slides were then blocked with 5% BSA for 1 h and underwent incubation using the primary antibody overnight at 4 °C, followed by incubation with Alexa Fluor 594 and/or 649-coupled secondary antibodies for 1 h at room temperature. Finally, the slides were stained utilizing 3,3-diaminobenzidine (DAPI, Sigma, USA, Cat# 28718-90-3) solution, mounted, and imaged with the utilization of an OLYMPUS confocal microscope (Olympus Optical Co., Ltd).

### Immunohistochemical (IHC) staining

Tissue chips containing ESCC and their marginal normal tissues were procured from Shanghai Outdo Biotech Co., Ltd., including 80 normal esophagus tissue and 105 ESCC tissue samples. Human ESCC biopsy samples (0.5–1 cm^3^) were fixed in 10% neutral buffered formalin, dehydrated, and immersed in paraffin, as per a previous report [19, 20]. Sections of 2.5-mm thickness were incubated in citrate buffer (pH 6.0) for 5 min at 120 °C, whereas 0.3% H_2_O_2_ for 10 min played a principal role in the blockade of the endogenous peroxidase. To enhance blockade of the nonspecific binding sites, the slides were subjected to 5% BSA in PBS for 30 min at 37 °C, accompanied by incubation with the applicable primary antibodies at 4 °C overnight and with horseradish peroxidase (HRP) anti-rabbit IgG or anti-mouse IgG antibodies for 1 h subsequently. The color was developed using the DAB Substrate kit (Cwbio, China, Cat# P10100). Upon rinsing the PBS, the tissue slices were counterstained with hematoxylin and eosin and visualized with the aid of a microscope. Major primary antibodies employed in the experiment included anti-human TAB182 antibody (1:150, Abcam, ab119429), β-catenin antibody (1:700, Abcam, ab196015), ALDH1A1 antibody (1:200, Proteintech, ab201986), and anti-CD133 antibody (1:50, Abcam, ab28364). Five randomly selected fields from every section were evaluated at the magnification of 20×. This investigation was approved by the Research Ethics Committee of The Affiliated Suzhou Hospital of Nanjing Medical University and all patients who participated offered informed consent.

### Luciferase reporter assay

The ESCC cell lines TE-10 or KYSE-150 were transfected with Top/Fop Flash plasmids utilizing Lipofectamine3000 Transfection Kit (Invitrogen, USA, Cat# L3000-015). The lysate cell’s luciferase activity was ascertained with the aid of a Dual-Luciferase Reporter Assay Kit (Promega, USA, Cat# E1910).

### Statistical analysis

The experimental data were statistically analyzed by Student’s t-test, paired Student’s t-test, or one-way ANOVA. All data are presented as mean ± S.E.M. *P < 0.05 and **P < 0.01 were statistically significant. Data from at least three independent experiments were utilized in all the cases. SPSS software package conducted all calculations. No randomization was followed and no blinding was carried out.

## Results

### TAB182 expression is upregulated in ESCC and promotes tumorigenesis in vitro and in vivo

**Fig. 1:**
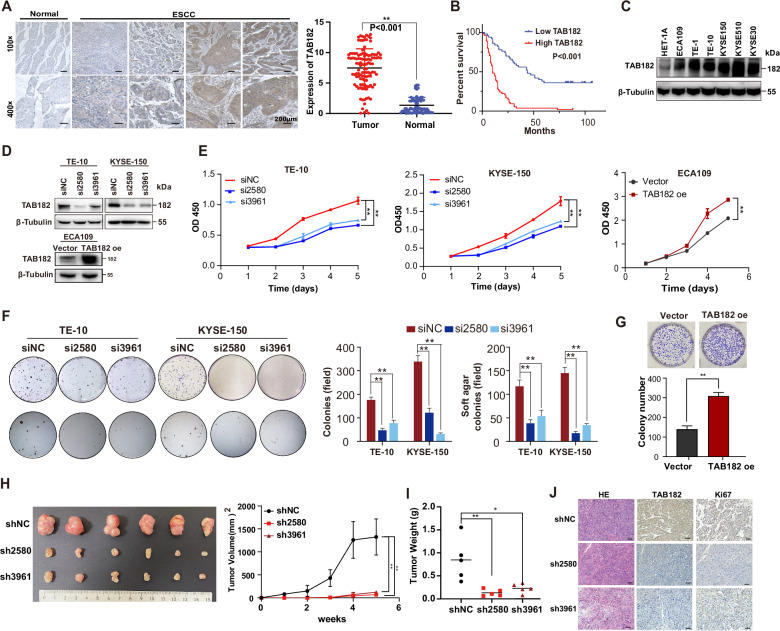
TAB182 expression is up-regulated in ESCC and promotes tumorigenesis in vitro and in vivo. **A** the expression of TAB182 in human ESCC tissues was determined by immunohistochemical staining (105 cases), bar, 200 μm. **B** the overexpression of TAB182 was closely associated with poor prognosis in ESCC patients. **C** Western blots results showed TAB182 expression levels in six ESCC cell lines and normal esophageal epithelial cells. **D**, protein levels of the TAB182 were detected by western blot in TAB182 knocked down and over-expressed ESCC cells 48 h post transfection. **E** Proliferation of TAB182 silenced and over-expressed cells was assessed by CCK8 assay 48 h post transfection. **F** soft agar and clonogenic assays were used to determine the clonogenic capacity of TE-10 and KYSE-150 cells 48 h post transfection. Clone numbers (>50 cells) in soft agar were determined after 2 weeks. **G**, clonogenic assay was performed to determine the colony formation ability of ECA109 cells. Clone numbers (>50 cells) were determined after 2 weeks. **H** subcutaneous xenograft tumors of the TAB182 silenced and control cells. xenograft tumors in mice followed up to 6 weeks. Data represented as mean ± SD (*n* = 6 mice per group). **I** Tumor weights are represented as the mean tumor weight ± SD. **J**, tumors from mice were stained with H&E to visualize tumor morphology. TAB182 and Ki67 were detected by immunohistochemical staining. *, *p* < 0.05; **, *p* < 0.01.

To explore the expression of TAB182 in ESCC, we performed immunohistochemical (IHC) staining on a tissue chip that included 105 ESCC samples as well as their paired normal tissues. The results of IHC revealed that TAB182 was expressed at a high level in the ESCC tumor tissues compared with that in the adjacent normal esophageal tissues (*P < 0.05; Fig. 1A). Furthermore, the expression levels of TAB182 were well linked to the differentiation status of ESCC tissues, with the highest TAB182 expression detected in the poorly differentiated ESCC tissues and the lowest TAB182 expression in well-differentiated ESCC tissues (Fig. S1A). Importantly, highly expressed TAB182 was associated with patients’ poor prognosis and shorter overall survival (Fig. 1B and Table 1). Similarly, the findings of western blot also showed that TAB182 is over-expressed in ESCC tissues (Fig. S1B). These results indicated the association between TAB182 and ESCC.

**Table 1:** The correlation between TAB182 expression and ESCC patients’ pathological features.

Characteristics	TAB182 expression		P value*
	low	high	
**Gender**			0.056
Male	23	54	
Female	14	14	
**Age**			0.267
≥60	10	14	
<60	29	24	
**Tumor invasion depth (T)**			0.002*
T1/T2	13	8	
T3/T4	22	62	
**Lymph node metastasis (N)**			0.0001*
N0	28	21	
N1-N3	12	45	
**TNM stage**			0.003*
I/II	35	38	
III/IV	8	31	

^*^chi-square test.

To examine the impacts of TAB182 on the tumorigenicity of ESCC cells, we firstly evaluated the protein levels of TAB182 in ESCC cell lines (ECA109, TE-1, TE-10, KYSE-150, KYSE-30 and KYSE-510). The findings of the western blot showed that TAB182 is also up-regulated in ESCC cells (Fig. 1C). Then, two siRNAs were used to knock down the expression of TAB182 in TE-10 and KYSE-150 cells, and TAB182 was exogenously over-expressed in ECA109 cells (Fig. 1D). Down-regulation of TAB182 in ESCC cells dramatically inhibited cell proliferation, while over-expression of TAB182 enhanced cell proliferation (Fig. 1E). Consistently, down-regulation of TAB182 impaired the colony formation ability and reduced the anchorage-independent cell growth in soft agar, while TAB182 over-expression enhanced the clonogenic capacity (Figs. 1F-G). As shown in Fig. 1H, silencing the expression of TAB182 in KYSE-150 cells (KYSE-150-shTAB182) remarkably inhibited the growth of tumors in nude mice compared with the controls (KYSE-150-shNC). Additionally, the tumor weight was heavier in the control group compared with that in the KYSE-150/shTAB182 group (Fig. 1I). Notably, immunohistochemical staining indicated that the tumors from KYSE-shTAB182-xenografted mice express lower TAB182 and Ki67 compared with tumors from the KYSE-shNC-xenografted mice (Fig. 1J). These findings suggest that highly expressed TAB182 exerts cancer-promotion function in ESCC.

### Down-regulation of TAB182 inhibits ESCC cells invasion and metastasis

**Fig. 2:**
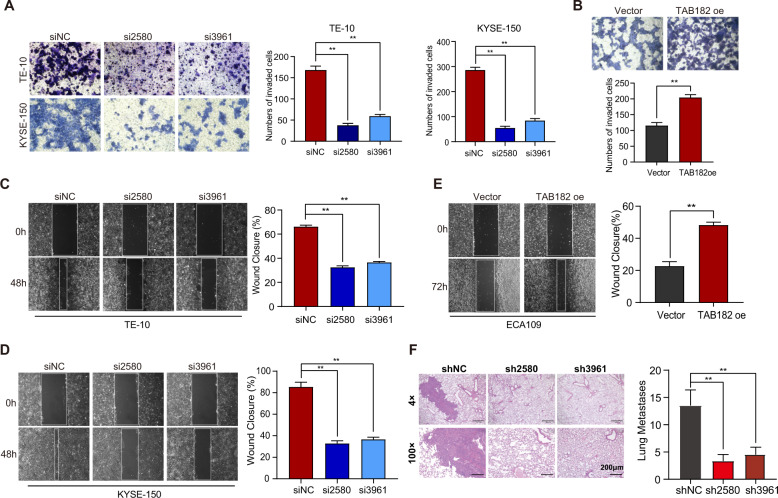
Down-regulation of TAB182 inhibits ESCC cells invasion and metastasis. **A** knockdown of the expression of TAB182 inhibited the invasion of TE-10 cells and KYSE-150 cells. **B** over-expression of TAB182 promoted the invasion of ECA109 cells. All groups started with the same cell numbers; cell numbers were counted after 24 h. **C**, **D** knockdown of the expression of TAB182 inhibited the migration of TE-10 cells and KYSE-150 cells. **E** over-expression of the expression of TAB182 enhanced the migration of ECA109 cells. The change in wound area was recorded daily with ImageJ software at 24, 48 and 72 h. The data were a representative of three repeats. Error bars indicate mean ± S.E.M. **P < 0.01, student t-test. **F** the metastasis of KYSE cells was assessed by the number of lung metastatic nodules 30 days after injection of cells through the tail vein of the mice. **, *p* < 0.01.

To ascertain the function of TAB182 in ESCC cell invasion as well as metastasis, we did wound-healing and transwell assays in vitro. The transwell assay showed that silencing TAB182 decreased the invasive ability of TE-10 and KYSE-150 cells, while TAB182 overexpression accelerated the invasion of ECA109 cells (Figs. 2A and B). Moreover, the results of the wound-healing assay also ascertained that knocking down of TAB182 expression remarkably reduced the migrative ability of TE-10 and KYSE-150 cells, however, increased TAB182 facilitated ECA109 cells migration (Figs. 2C–E). In addition, KYSE-150-shNC and KYSE-150-shTAB182 cells were injected into the tail vein of nude mice to verify the impact of TAB182 on ESCC cells metastasis in vivo. Stable knockdown of TAB182 resulted in a reduction in lung colonization following the tail vein injection (Fig. 2F). These findings suggest that silencing TAB182 in ESCC cells reduces the capacity of metastatic ESCC cells to migrate and invade, which is consistent with our findings in Table 1, which showed that TAB182 expression was strongly linked to tumor invasion depth (*P* = 0.002), lymph node metastasis (*P* = 0.0001), and TNM stage (*P* = 0.003).

### TAB182 regulates the β-catenin signaling pathway by affecting the nuclear translocation of β-catenin

**Fig. 3:**
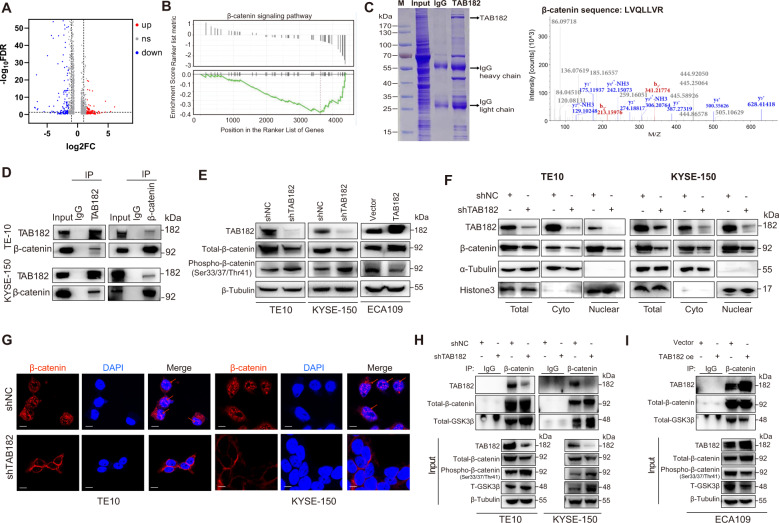
TAB182 regulates the β-catenin signaling pathway via affecting the nuclear translocation of β-catenin. **A** volcano plot showing differential expressed genes between shNC and shTAB182 TE-10 cells. **B** RNA transcriptome data was reanalyzed using Gene Set Enrichment Analysis. NES = −1.52, *P* < 0.05. **C** Coomassie Blue-stained SDS-PAGE gel of co-immunoprecipitation products of TAB182. The MS/MS spectra show the presence of peptide peaks corresponding to the β-catenin sequence (LVQLLVR). **D** co-immunoprecipitation was performed by using TAB182 and β-catenin antibody, and the IP product was analyzed by immunoblotting. **E** western blotting analysis of the indicated total β-catenin and phosphorylated β-catenin proteins. **F** western blotting analysis of nuclear expression of β-catenin in TAB182 knockdown and overexpressed cells. **G** immunofluorescence staining of nucleus location of β-catenin in TE-10-shNC and TE-10-shTAB182 cells, KYSE-150-shNC and KYSE-150-shTAB182 cells. **H, I** immunoprecipitation detection of interaction between β-catenin and GSK3β in TAB182 decreased and overexpressed ESCC cells.

To further determine the underlying mechanism of the modulatory effects of TAB182 in ESCC cells, we used RNA-seq analysis to determine the differential gene expression profiles between TE-10-shTAB182 and TE-10-shcon cells. With ≥1.5-fold changes as a cutoff threshold, 756 genes were found to be downregulated and 509 genes upregulated (Fig. 3A and Table S2). Importantly, GO pathway analysis showed that the expression of various genes is important for tumorigenesis, stemness, EMT, and metastasis was remarkably downregulated in TE-10-shTAB182 cells in contrast with the control cells (Fig. S1C and D). Meanwhile, GSEA analysis revealed that the expression of TAB182-regulated genes is strongly associated with the β-catenin signaling pathway (Fig. 3B). Interestingly, we simultaneously performed co-immunoprecipitation combined with mass spectrometry and found that TAB182 could interact with β-catenin in ESCC cells (Fig. 3C and Table S3). Furthermore, the link between TAB182 and β-catenin was confirmed by western blot following IP in ESCC cells (Fig. 3D). These findings motivated us to investigate how TAB182 affects the β-catenin signaling by binding with β-catenin. As shown in Fig. 3E, inhibition of TAB182 expression decreased the total protein level of β-catenin but increased the phosphorylated β-catenin (Ser33/37/Thr41), while over-expression showed a converse effect.

Generally, decreased Ser33/37/Thr41 phosphorylation that is catalyzed by GSK3β prevents β-catenin from subsequent ubiquitination-mediated degradation, and promotes the nucleus-translocation of β-catenin. As expected, TAB182 silencing significantly lowered the protein level of β-catenin in the nucleus of ESCC cells, while over-expression of TAB182 elevated the nucleus distribution of β-catenin (Fig. 3F). Also, the immunofluorescence staining showed the same result (Fig. 3G). Of importance, downregulation of TAB182 led to enhanced interaction between β-catenin and GSK3β, while TAB182 over-expression impaired their interaction in ESCC cells (Figs. 3H, I). These data indicated that TAB182 might contribute to the progression of ESCC through regulating the nucleus translocation of β-catenin.

### TAB182 regulates nucleus translocation of *β*-*catenin* via interacting with FHL2

**Fig. 4:**
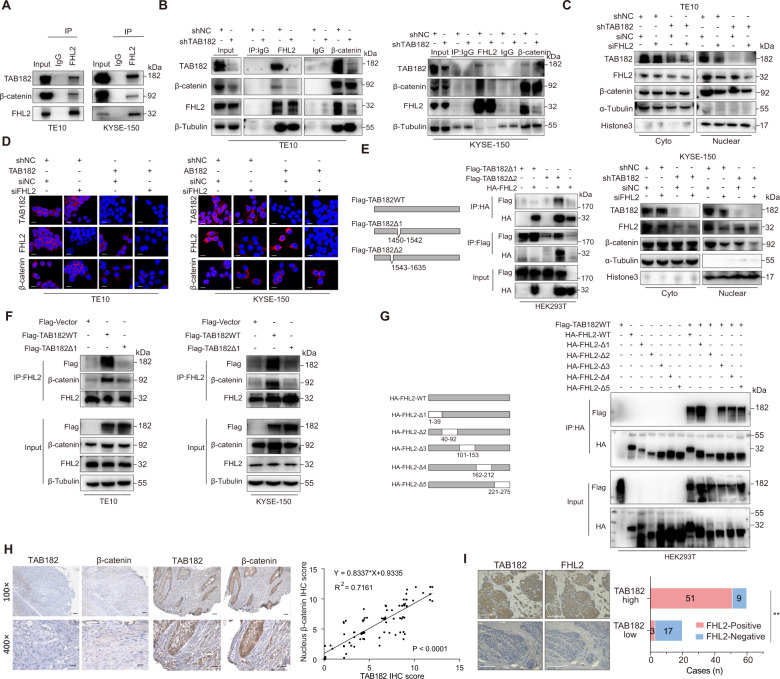
TAB182 regulates nucleus translocation of β-catenin via interacting with FHL2. **A** the interaction between TAB182, FHL2 and β-catenin were determined by immunoprecipitation followed by immunoblotting analysis. **B** the interaction between FHL2 and β-catenin in TAB182 silenced cells was detected by immunoprecipitation. **C** the distribution of β-catenin in TE-10 and KYSE-150 cells was evaluated by fractionation followed by western blot analysis. **D** the distribution of β-catenin in TE-10 and KYSE-150 cells was evaluated by immunofluorescence staining. **E** determining the interaction domain of TAB182 with FHL2 in HEK293T cells by immunoprecipitation assay. HEK293 cells were transfected with Flag-TAB182 or HA-FHL2 plasmid for 48 h, followed by co-IP and western blot. **F** the interaction between FHL2 and β-catenin was determined in the TAB182-Δ1cells and TAB182-WT cells by immunoprecipitation assay in TE-10 and KYSE-150 cells. **G** determining the interaction domain of FHL2 with TAB182. Co-immunoprecipitation assay showed the ability of five mutants of FHL2 binds to TAB182 by immunoprecipitation assay. HEK293 cells were transfected with Flag-TAB182 or HA-FHL2 plasmid for 48 h, followed by co-IP and western nlot. **H** the correlation between TAB182 and nucleus β-catenin expression in ESCC samples was analyzed by immunohistochemistry and Pearson correlation analysis (r = 0.7161, *P* < 0.0001). **I** representative immunohistochemistry-stained images of ESCC tissues using the anti-TAB182 and anti-FHL2 antibodies. **, *p* < 0.01.

The above findings motivated us to investigate the underlying molecular mechanism through which TAB182 regulates β-catenin nuclear translocation in ESCC cells. Our previous study revealed that TAB182 interacts with FHL2, which has been reported to mediate β-catenin nucleus translocation and TCF/LEF transcription [21]. To ascertain if FHL2 is involved in TAB182 mediated nucleus translocation of β-catenin, a co-immunoprecipitation assay was performed. Co-IP results showed that TAB182, FHL2, and β-catenin could co-exist in a protein complex in ESCC and HEK293T cells (Fig. 4A and Fig. S2A). Moreover, knockdown of TAB182 impaired the interaction of FHL2 and β-catenin (Fig. 4B). Fractionation assay, immunofluorescence, and IHC staining showed that knockdown of FHL2 significantly attenuated the nucleus translocation of β-catenin and further exacerbated the reduced β-catenin nucleus translocation caused by TAB182 knockdown in ESCC cells (Figs. 4C, D and Fig. S2B).

The RXXPDG motif of TAB182 has been reported to be important for TAB182 interaction with other binding proteins [22]. We next constructed the TAB182 wild-type (Flag-TAB182-WT) and mutant (Flag-TAB182-Δ1 and Flag-TAB182-Δ2) expression plasmids. Co-immunoprecipitation demonstrated that TAB182-Δ1 was unable to interact with FHL2 while TAB182-Δ2 was able to bind to FHL2 (Fig. 4E). Furthermore, it was found that in TAB182-Δ1 expressed ESCC cells, the interaction between FHL2 and β-catenin was attenuated (Fig. 4F). To determine the binding sites on FHL2, we generated different truncated FHL2 expression vectors. Co-immunoprecipitation analysis demonstrated that FHL2-Δ2 was unable to interact with TAB182 (Fig. 4G), which indicated that FHL2 interacted with TAB182 dependent on the 40-92aa domain. Next, we evaluated the association between TAB182 and nucleus β-catenin or FHL2 in ESCC tissues. Immunohistochemistry analysis of ESCC samples affirmed that TAB182 expression was positively related to the FHL2 and nucleus β-catenin expression (Figs. 4H and I). Taken together, these results demonstrate that TAB182 mediates promotion of β-catenin nucleus translocation is at least partially dependent on FHL2 in ESCC cells.

### Silencing TAB182 expression significantly reduces the stemness of ESCC cells

**Fig. 5:**
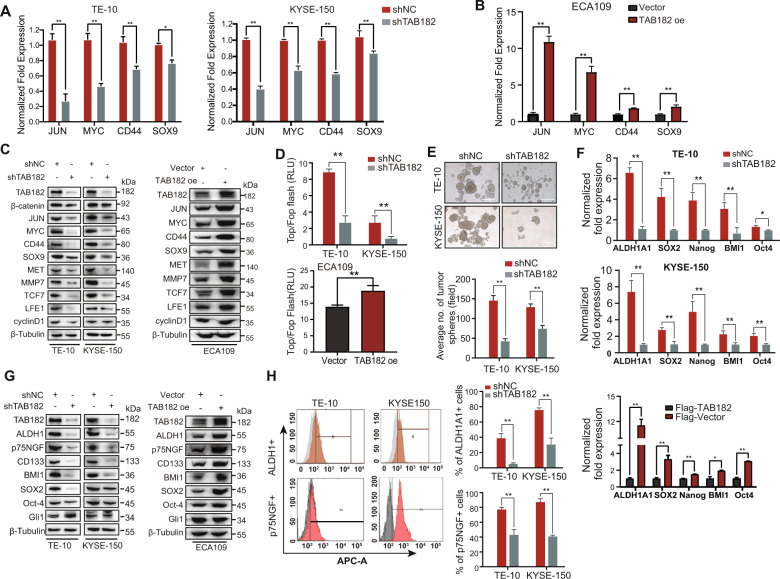
Silencing TAB182 expression significantly reduces the stemness of ESCC cells. **A** The DNA microarray results were further confirmed using real-time quantitative PCR (QT-PCR). **B** expression levels of ALDH1A1, SOX2, Nanog, BMI1 and Oct4 were quantitatively measured using QT-PCR. **C** the expression of the Wnt/β-catenin signaling pathway downstream genes, as analyzed by western blot. **D** TOP/FOP luciferase reporter assays were performed in KYSE-150-shNC and KYSE-shTAB182 cells. Luciferase activity was measured 48 h after co-transduced with TOP/FOP flash plasmid. **E** the formation of tumor spheres was performed to evaluate the stemness of KYSE-150 and TE-10 cells. Average number of tumor spheres formed with knockdown of TAB182 were counted at 96 h. **F**, expression levels of ALDH1A1, SOX2, Nanog, BMI1 and Oct4 were quantitatively measured using qRT-PCR. **G** the protein levels of CD133, ALDH1A1, BMI1, p75NGF and SOX2 were detected in TE10, KYSE-150 and ECA109 cells by immunoblotting. **H** the percentage of ALDH1A1 positive TE10 and KYSE-150 cells was analyzed using FACS. *, *p* < 0.05; **, *p* < 0.01.

To further verify whether TAB182 activates β-catenin signaling, we evaluate the expression of multiple representative target genes of canonical β-catenin signaling. As exhibited in Fig. 5A, the expression of JUN, CD44, c-Myc (MYC), and SOX9 was decreased following knockdown of TAB182, but increased in TAB182 over-expressed cells (Fig. 5B). Additionally, the protein levels of the β-catenin downstream target genes JUN, MYC, CD44, SOX9, and MMP7 were altered accordingly (Fig. 5C). Consistently, knockdown of TAB182 significantly reduced the Top/Flash reporter activity in ESCC cells as expected (Fig. 5D). There is now substantial evidence supporting the function of the β-catenin signaling pathway in the sustenance of the stemness of cancer cells in ESCC. To determine whether TAB182 influences the stemness of ESCC cells, we performed sphere-formation assays. Compared with control cells, the TAB182-knockdown cells exhibited a considerably lower tumor sphere-forming ability (Fig. 5E). Next, we investigated the impact of TAB182 knockdown on the transcription of stemness-related markers. As predicted, TAB182 knockdown remarkably attenuated the transcription of stemness-related genes, e.g., ALDH1A1, BMI1and Oct-4, as well as the genes encoding surface markers of cancer stem cells such as p75NGF, while over-expression of TAB182 showed conversed effect (Fig. 5F). Consistently, the result of the western blot showed the same alterations (Fig. 5G). These findings were further corroborated by flow cytometry analysis showing that the counts of ALDH1A1 and p75NGF positive cells are remarkably reduced in the shTAB182 population (Fig. 5H). Moreover, we discovered a substantial positive connection between TAB182 expression and ALDH1A1 localization in 105 ESCC samples (Fig. S2D, E). Conclusively, these findings strongly imply that ESCC cells with a high expression of TAB182 are more likely to be associated with more aggressive cancer features.

### TAB182’s function depends on the 484-514aa domain

**Fig. 6:**
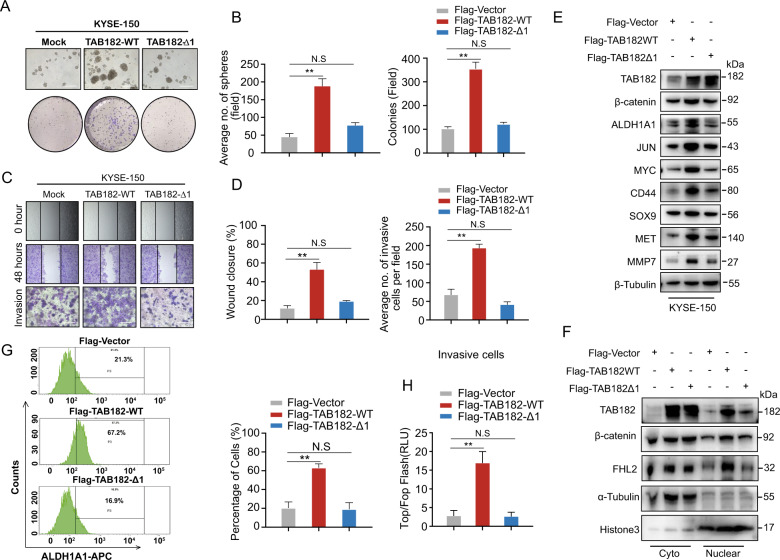
TAB182’s function depends on the 484-514aa domain. **A**, **B** Sphere formation and colony formation assays were conducted to evaluate the stemness and clonogenic ability of TAB182-Δ1 and wild type-TAB182 expressed cells. **C**, **D** wound healing and transwell assays were performed to examine cell migration and invasion ability. **E** western blot was sued to determine β-catenin downstream target genes expression in TAB182-Δ1 and TAB182-WT expressed cells. **F** distribution of TAB182, β-catenin and FHL2 protein was determined by western blot. **G** percentage of ALDH1A1 expressed cells was determined by FACS. **H** TOP/FOP luciferase assay was used to examine the β-catenin activation in the TAB182-WT and TAB182-Δ1 expressed cells. Luciferase activity was measured 48 h after co-transduced with TOP/FOP flash plasmid. Three individual experiments were performed, and error bars of the data indicate mean ± S.E.M., **P < 0.01.

As the 484-514aa domain is essential for TAB182’s interaction with FHL2, we further evaluated whether this domain is required for TAB182 to be functional in ESCC cells. Our results showed that wild-type TAB182 could facilitate the growth of ESCC cell oncospheres and enhance the ability of colony formation, but the mutant TAB182-Δ1 cells had no such impact (Figs. 6A and B). The invasion and wound healing assays showed that wild-type TAB182, but not TAB182-Δ1, promoted the invasive and migration ability of KYSE-150 cells (Fig. 6C and D). Moreover, the protein levels of several β-catenin downstream targets were up-regulated in wild-type TAB182 cells compared to that in TAB182-Δ1 cells (Fig. 6E). In addition, nucleus accumulation of β-catenin and FHL2 was increased in TAB182-WT cells but not in the mutant TAB182-Δ1 cells (Fig. 6F). The over-expression of wild-type TAB182, not TAB182-Δ1 was also found to increase the ALDH1A1 + cell subpopulation (Fig. 6G). Consistently, the TOP flash reporter activity was remarkably increased in wild-type TAB182 cells in contrast with that in control and TAB182-Δ1 cells (Fig. 6H). These findings indicate that TAB182 exerts oncogenic function in ESCC cells in a 484-514aa domain-dependent manner.

### TAB182 is a novel regulator of the FHL2-β-catenin axis

**Fig. 7:**
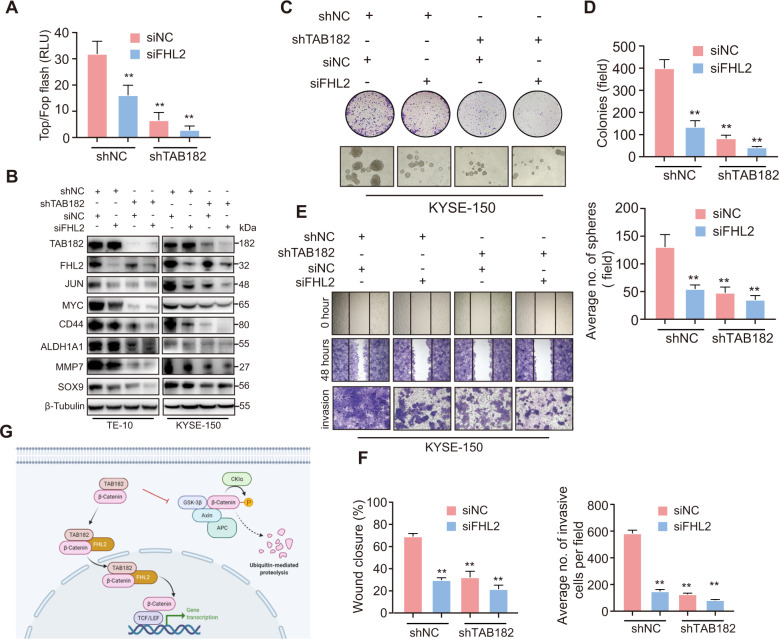
TAB182 is a novel regulator of the FHL2-β-catenin axis. **A** TOP/FOP luciferase assay was used to examine the β-catenin activation in KYSE-150 cells. **B** protein expression of the β-catenin target genes was determined by western blot. **C**, **D** cell clonogenic ability and clonal sphere formation ability of TAB182-Δ1 and TAB182-WT cells was evaluated. **E**, **F** migratory and invasive capabilities of TAB182-Δ1 and TAB182-WT cells were evaluated using transwell and wound healing assays. **G** schematic depiction of the mechanisms how TAB182 act as a novel activator of the FHL2-β-catenin axis in ESCC cells.

To verify the presence of the TAB182-FHL2 axis in ESCC cells, the expression of FHL2 was knocked down by siRNA in ESCC cells. Knockdown of FHL2 expression led to a remarkable decrease in TCF/LEF promoter activity in shNC cells, and the activity of TCF/LEF promoter was even lower in FHL2 and TAB182 both down-regulated cells (Fig. 7A). Comparable results were observed in the western blotting examining the expression of the β-catenin downstream target genes (Fig. 7B). Meanwhile, the clone formation and sphere formation assays examining tumorigenesis and stemness (Figs. 7C and D), and the wound healing and invasion assays analyzing migration and invasive potential (Figs. 7E and F) showed the same findings. Together, these findings reinforce the concept that TAB182 is a new activator of the FHL2-β-catenin axis in ESCC cells (Fig. 7G).

## Discussion

One of the most frequently altered pathways in ESCC is the Wnt/β-catenin signaling pathway [23–25]. This pathway exerts a fundamental function in development by modulating various cellular processes, for instance, proliferation, migration, cell fate determination, cancer cell stemness and embryogenesis as well as tumorigenesis. Nucleus accumulation of β-catenin, a hallmark of β-catenin signaling activation, replaces transcription inhibitors to bind to the TCF/LEF transcription factor family, thus activating target gene expression and promoting above mentioned cellular processes [5, 26, 27]. The β-catenin signaling pathway is known to trigger tumorigenesis and stemness in ESCC cells but the underlying mechanisms are yet uncertain.

In this study, we manifested that TAB182 is expressed at a high level in ESCC tissues and related to patients’ poor prognosis, indicating that TAB182 is likely involved in the progression of ESCC. Loss-of-function investigations, on the other hand, revealed the vital functions of TAB182 in promoting tumorigenesis and invasiveness of ESCC cells in addition to the maintenance of their stemness. Evidence from in vivo studies confirmed that TAB182 promotes ESCC aggressiveness and metastasis. Importantly, we demonstrated that the tumors with a greater percentage of high-TAB182-expressing cells, which are also exhibiting highly expressed ALDH1A1, are linked to a poor prognosis among ESCC patients. As a member of the poly (ADP-ribose) polymerase (PARP) superfamily and the target protein of Tankyrase, TAB182 was previously reported to interact with DNAPKcs and involve in the DNA damage repair reaction [8]. We have recently reported that TAB182 heightens the radioresistance of ESCC cells by controlling the G2-M checkpoint via its interaction with FHL2 [9]. In this context, Ohishi et al. affirmed that TAB182 regulates the invasion of pancreatic malignant cells by binding to the actin-capping protein CapZA2 and enhancing its’ interaction with the cytoskeleton [28], suggesting that TAB182 may act differently in different types of cancer cells.

Tumorigenesis and tumor malignant nature are often associated with cancer cell stemness. ESCC cells with cancer stem cells characteristics have been considered to more inclined to distant metastasis, resulting in poor prognosis of the patients [29]. Here, we discovered that TAB182 promotes the stemness and tumorigenicity of ESCC cells by triggering the nucleus translocation of β-catenin to stimulate the β-catenin pathway, which leads to the activated transcription of JUN, MYC, ALDH1A1, CD44, and CD133. In accordance with our findings, Huang revealed that the activation of β-catenin signaling enhances the androgen-independent self-renewal of ESCC cells with stem cell-like characteristics [30]. While the molecular processes underlying the modulation of β-catenin by TAB182 in ESCC remain to be further elucidated, we discovered that TAB182 physically interacts with β-catenin and prevents it from being phosphorylated by GSK3β and ubiquitination-mediated degradation. TAB182 further recruits FHL2 and forms a TAB182-FHL2-β-catenin complex allowing for efficient FHL2-mediated β-catenin nucleus translocation by enhancing the association between FHL2 and β-catenin, and this is dependent on the RXXPDG motif of TAB182 in ESCC cells. These findings indicated that TAB182 may serve as a crucial malignant factor and novel stemness-related modulator via the TAB182-FHL2-β-catenin axis in ESCC.

## Conclusions

In summary, our study reveals the novel oncogenic role of TAB182 in ESCC and avail useful insights into a probable function of the TAB182/FHL2/β-catenin molecular axis in ESCC cell stemness, invasiveness, and tumorigenicity. Of clinical relevance, TAB182 might function as a potential diagnostic marker for ESCC. Additionally, targeting the TAB182/FHL2/β-catenin can be a new avenue for the development of therapeutics against ESCC.

## Supplementary information

Supplementary information

Supplementary Figure 1

Supplementary Figure 2

Supplementary Table 1

Supplementary Table 2

Supplementary Table 3

Full Western Blots

Reproducibility checklist

## Data Availability

The dataset(s) supporting the findings of this study are included within the article.

## Materials availability

Requests for materials should be addressed to J-DZ and M-S.
