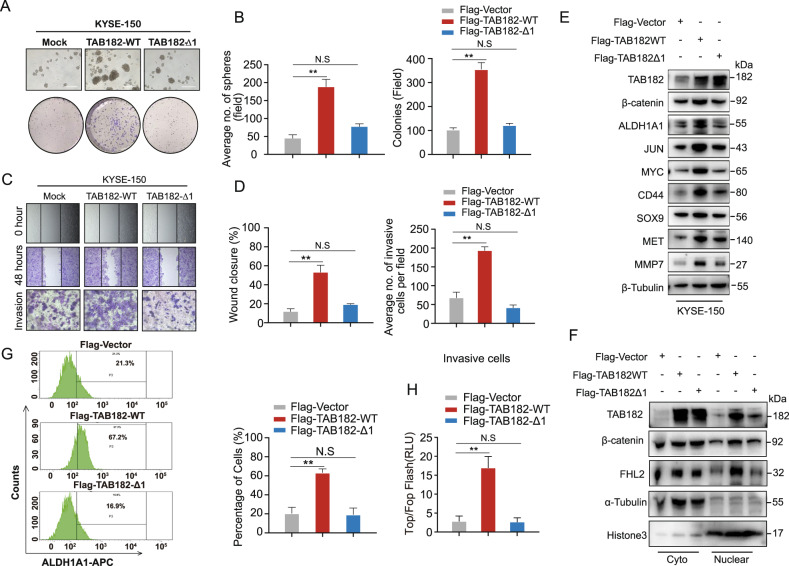# Correction: TAB182 aggravates progression of esophageal squamous cell carcinoma by enhancing β-catenin nuclear translocation through FHL2 dependent manner

**DOI:** 10.1038/s41419-023-05799-9

**Published:** 2023-04-14

**Authors:** Aidi Gao, Zhenzi Su, Zengfu Shang, Chao He, Dongliu Miao, Xiaoqing Li, Shitao Zou, Weiqun Ding, Yue Zhou, Ming Sun, Jundong Zhou

**Affiliations:** 1 Suzhou Cancer Center Core Laboratory, The Affiliated Suzhou Hospital of Nanjing Medical University, Suzhou, Jiangsu P.R. China; 2 The Affiliated Suqian Hospital of Xuzhou Medical University and Nanjing Drum Tower Hospital Group Suqian Hospital, Suqian, Jiangsu P.R. China; 3 School of Radiation Medicine and Protection, Medical College of Soochow University, Suzhou, China; 4 Department of Pathology, University of Oklahoma Health Science Center, Oklahoma City, OK USA; 5 Department of Thoracic Surgery, First Affiliated Hospital of Nanjing Medical University, Nanjing, China; 6 Suzhou Cancer Center Core Laboratory, The Affiliated Suzhou Hospital of Nanjing Medical University, Suzhou Municipal Hospital, Gusu School, Baita west road #16, 215001 Suzhou, China

**Keywords:** Oncogenes, Cell growth

Correction to: *Cell Death and Disease* 10.1038/s41419-022-05334-2, published online 26 October 2022.

The original version of this article contained errors in figures 2 and 6. The corrected figures can be found below. The original article has been corrected.

Fig. 2.
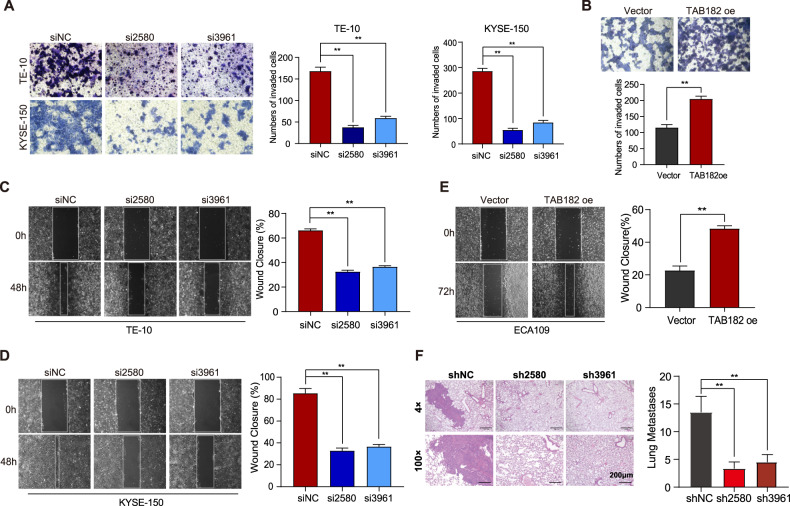
Fig. 6.