# Correction: TAB182 aggravates progression of esophageal squamous cell carcinoma by enhancing β-catenin nuclear translocation through FHL2 dependent manner

**DOI:** 10.1038/s41419-025-08077-y

**Published:** 2025-12-23

**Authors:** Aidi Gao, Zhenzi Su, Zengfu Shang, Chao He, Dongliu Miao, Xiaoqing Li, Shitao Zou, Weiqun Ding, Yue Zhou, Ming Sun, Jundong Zhou

**Affiliations:** 1 Suzhou Cancer Center Core Laboratory, The Affiliated Suzhou Hospital of Nanjing Medical University, Suzhou, Jiangsu PR China; 2 The Affiliated Suqian Hospital of Xuzhou Medical University and Nanjing Drum Tower Hospital Group Suqian Hospital, Suqian, Jiangsu PR China; 3 School of Radiation Medicine and Protection, Medical College of Soochow University, Suzhou, China; 4 Department of Pathology, University of Oklahoma Health Science Center, Oklahoma City, OK USA; 5 Department of Thoracic Surgery, First Affiliated Hospital of Nanjing Medical University, Nanjing, China; 6 Suzhou Cancer Center Core Laboratory, The Affiliated Suzhou Hospital of Nanjing Medical University, Suzhou Municipal Hospital, Gusu School, Suzhou, China

**Keywords:** Oncogenes, Cell growth

Correction to: *Cell Death & Disease* 10.1038/s41419-022-05334-2, published online 26 October 2022

While the first author, Dr. Aidi Gao was conducting a follow-up study, she recently re-checked the original data and figures from this publication and discovered an error in the Figure 1J. Due to a mistake during the image formatting process, the labels for the horizontal and vertical annotations in Figure 1J panels were swapped by Gao. As a result, two IHC images of the same group were used, and one was incorrectly labeled as representing a different marker.

**Amended Figure1J** 
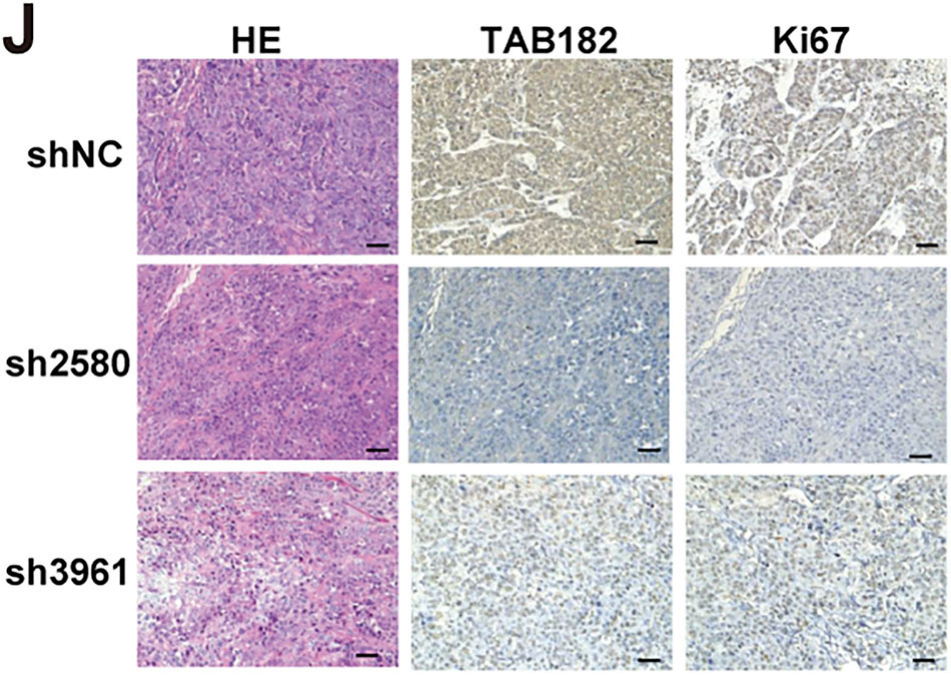


**Original Figure 1J** 
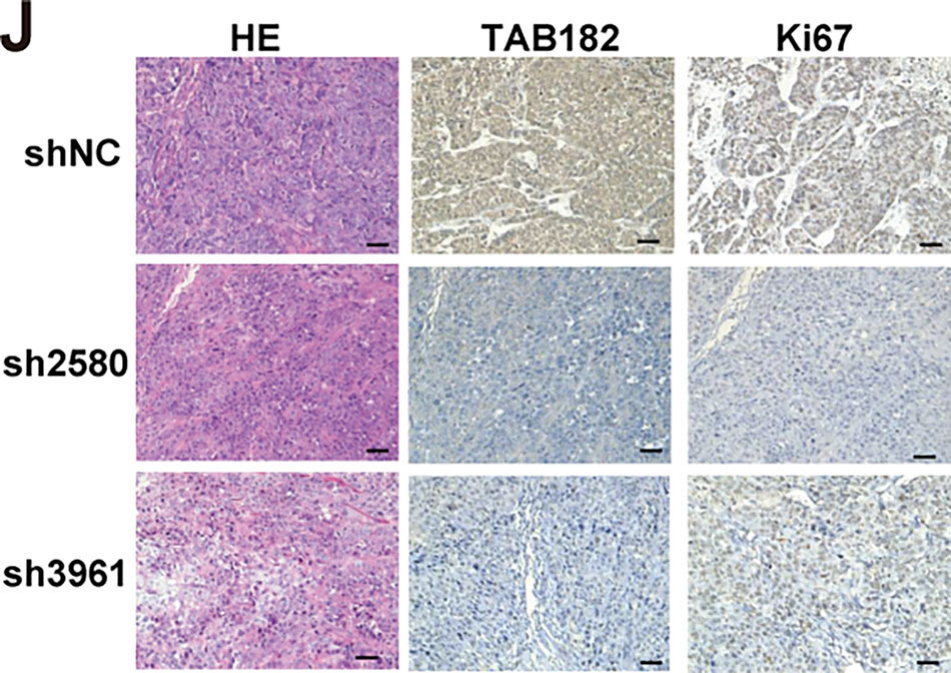


**Original Figure 1** 
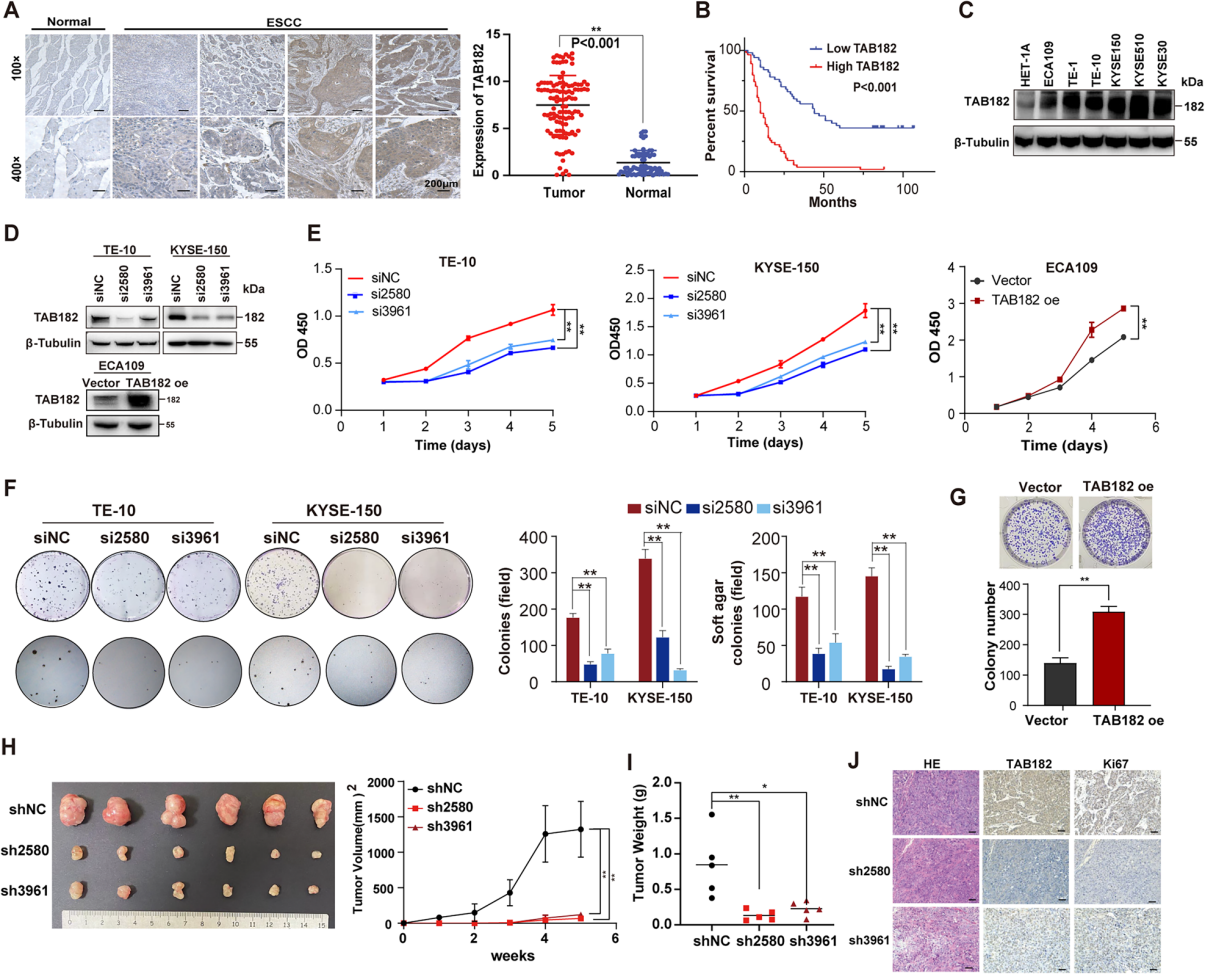


The original article has been corrected.

