# Supplementary material for: TAB182 aggravates progression of esophageal squamous cell carcinoma by enhancing β-catenin nuclear translocation through FHL2 dependent manner
Source: Cell Death Dis. 2022 Oct 26;13(10):900. doi: 10.1038/s41419-022-05334-2 (PMC9606255; doi:10.1038/s41419-022-05334-2)
Supplement: Supplementary file 1 — Supplementary information [file 41419_2022_5334_MOESM1_ESM.docx]

**Supplementary Figure 1.** TAB182 is over-expressed in ESCC and activated β-catenin pathway. A, immunohistochemical analysis of ESCC tissues determining TAB182 expression levels and correlation with the differentiation. B, the protein levels of TAB182 in 7 pairs of human ESCC tumor tissues (T) and adjacent esophageal normal tissues (NT) were determined by immunoblotting. C, heatmaps of differentially expressed genes between in TAB182 down-regulated cells. D, KEGG pathway analysis revealed that the TAB182-regulated genes was associated with β-catenin signaling pathway.

**Supplementary Figure 2.** TAB182 regulated nucleus translocation of β-catenin via interacting with FHL2. A, co‐IP of Flag-TAB182 with HA-FHL2 in 293T cells. B, immunohistochemistry was used to determine the cellular localization of β-catenin in ESCC cells. C, correlations between TAB182 expression and ALDH1A1 localization in ESCC. D, positive correlation between TAB182 and ALDH1A1 expression in ESCC tissue samples. R^2^=0.6432, p<0.001. E, released wnt5A in cell supernatants were determined by ELISA in TE-10 and KYSE-150 cells.

**Supplementary Table 1.** The sequence of primers and siRNAs, and antibody information.

**Supplementary Table 2.** Differentially expressed genes in TAB182 down-regulated cells.

**Supplementary Table 3.** Results of MS analysis.
