# Supplementary figures and images for: TAB182 aggravates progression of esophageal squamous cell carcinoma by enhancing β-catenin nuclear translocation through FHL2 dependent manner
Source: Cell Death Dis. 2022 Oct 26;13(10):900. doi: 10.1038/s41419-022-05334-2 (PMC9606255; doi:10.1038/s41419-022-05334-2)

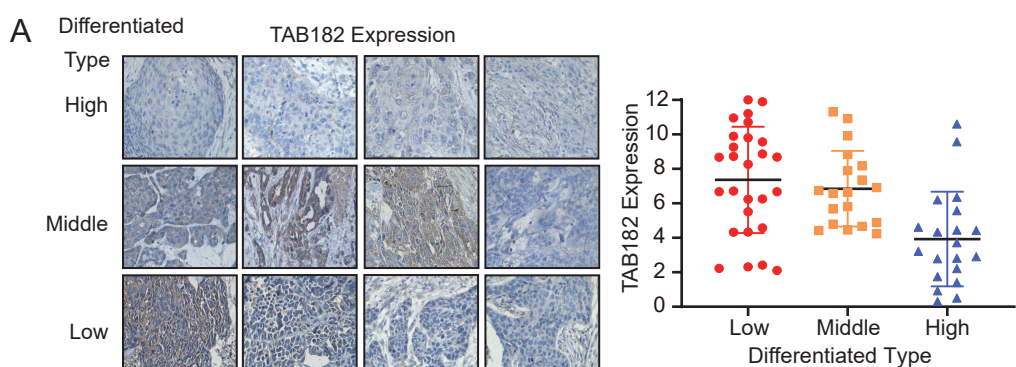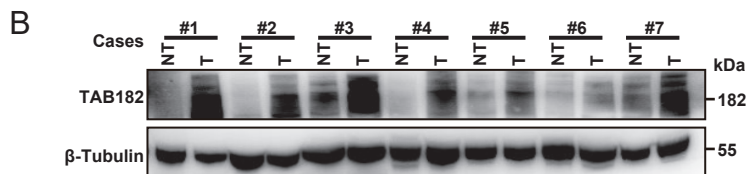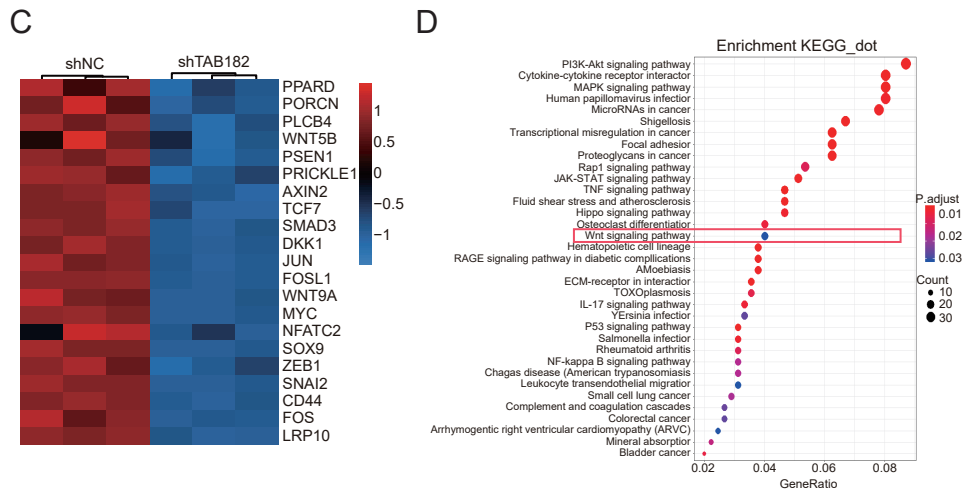

Supplement: Supplementary file 2 — Supplementary Figure 1 [file 41419_2022_5334_MOESM2_ESM.pdf]

A

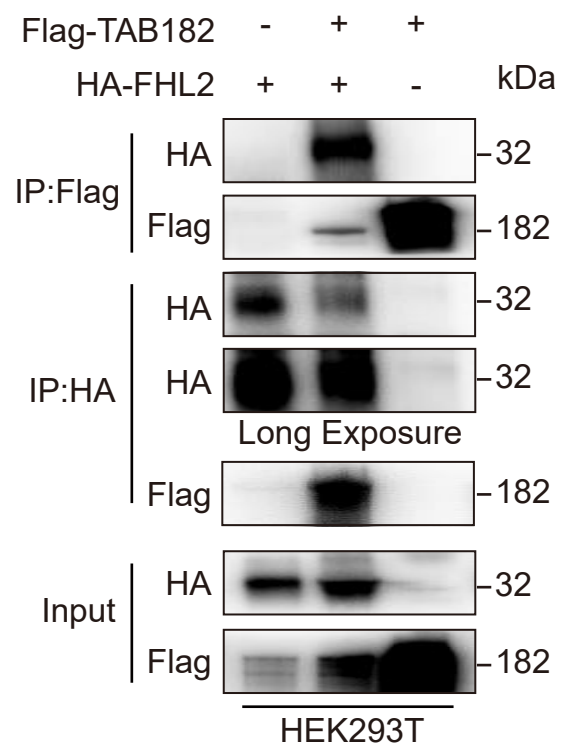

B

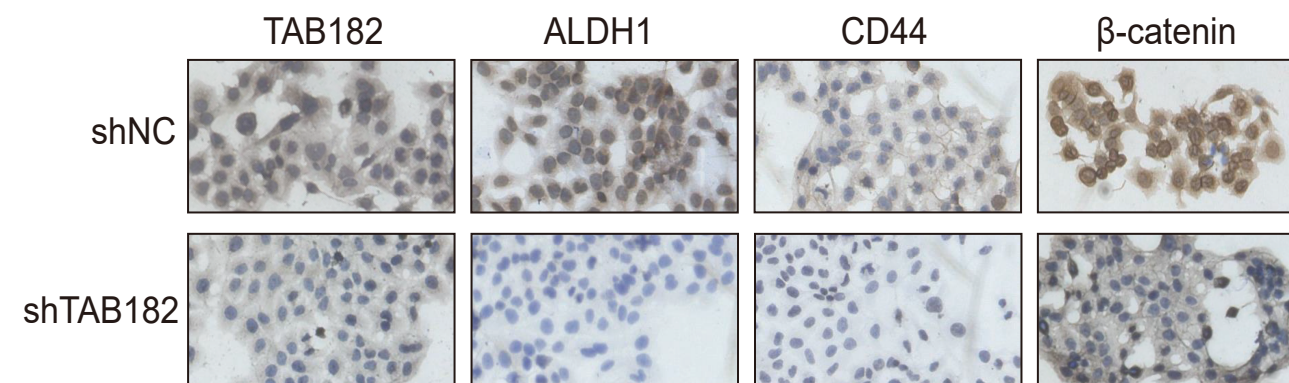

C

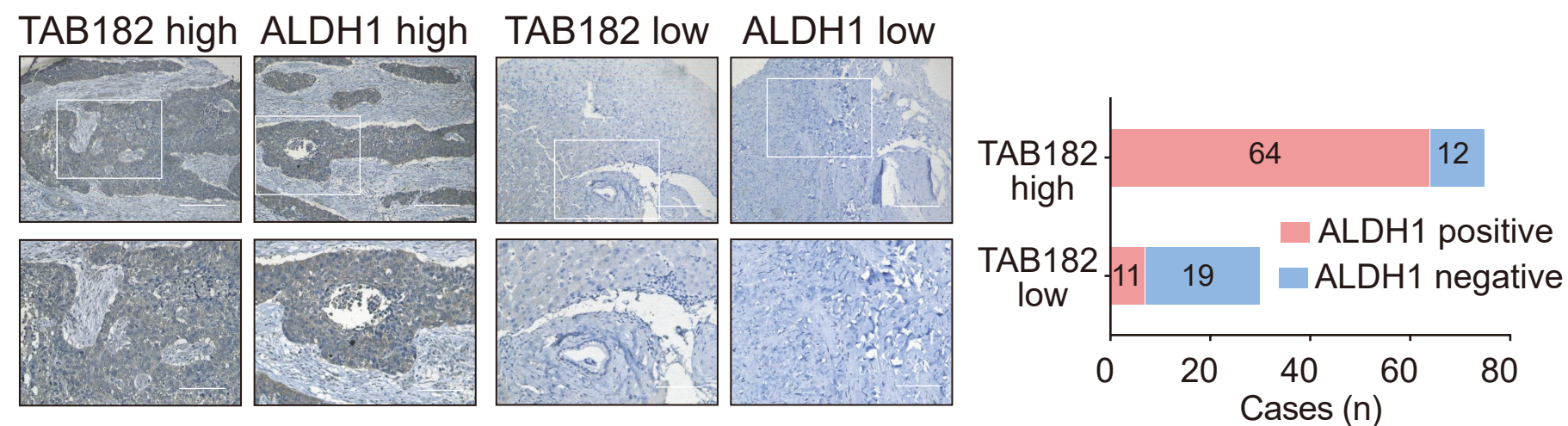

D

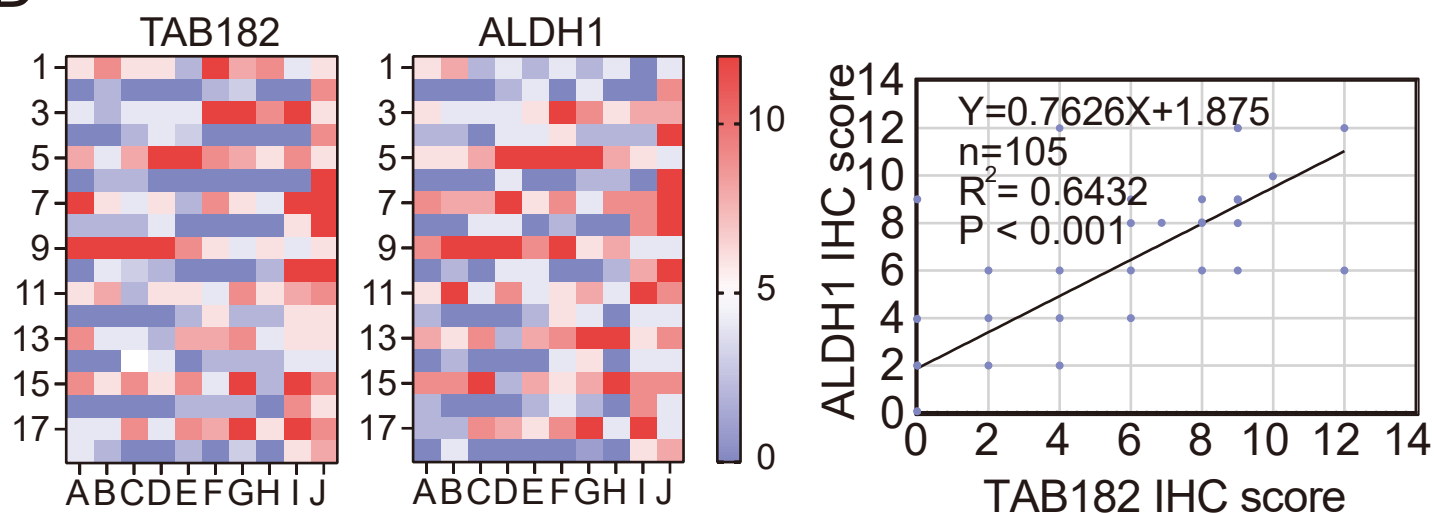

Supplement: Supplementary file 3 — Supplementary Figure 2 [file 41419_2022_5334_MOESM3_ESM.pdf]

Figure 1

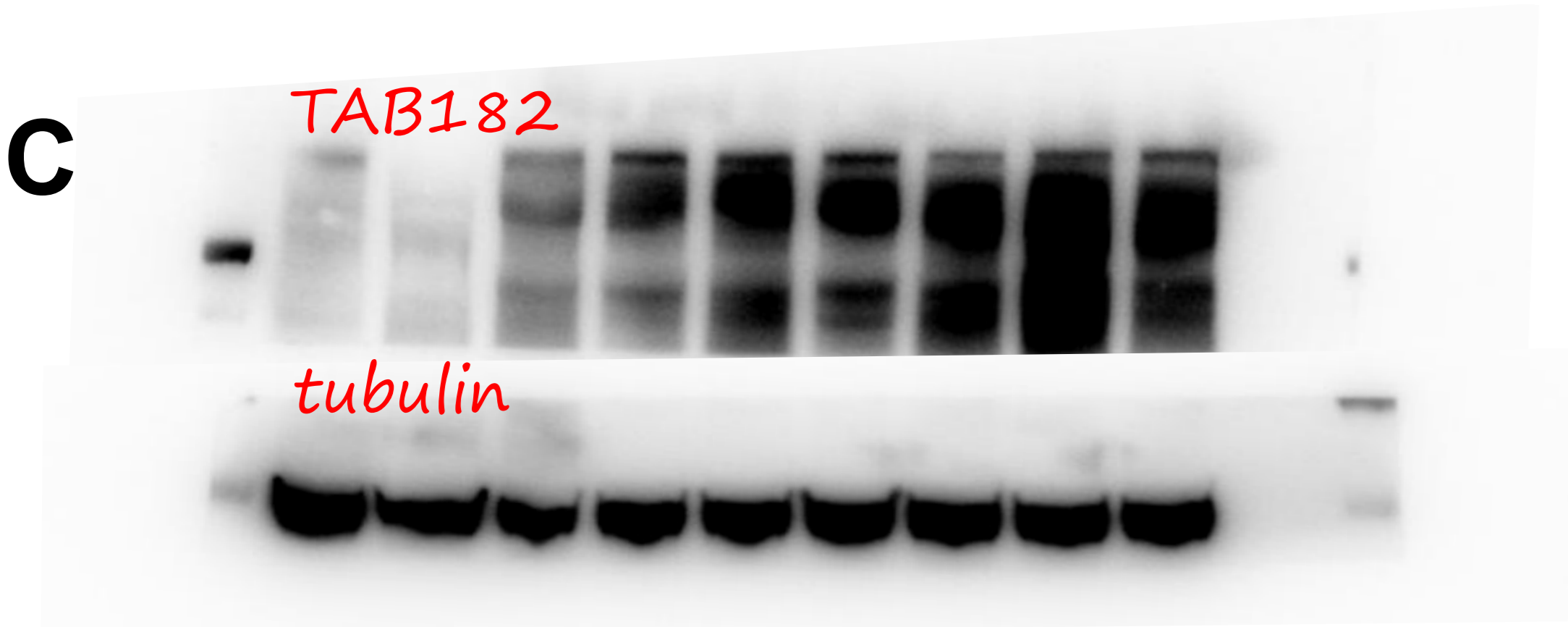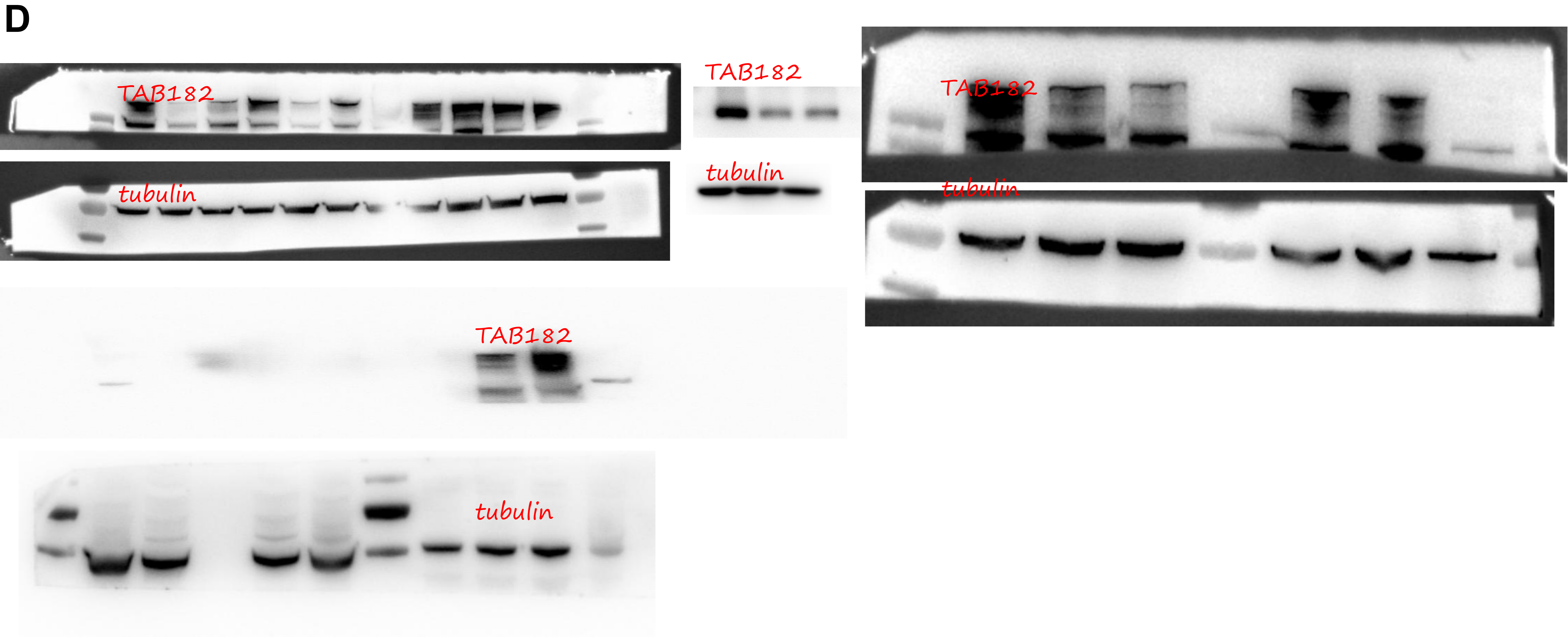

. .  
. . . . .



Figure 3

H

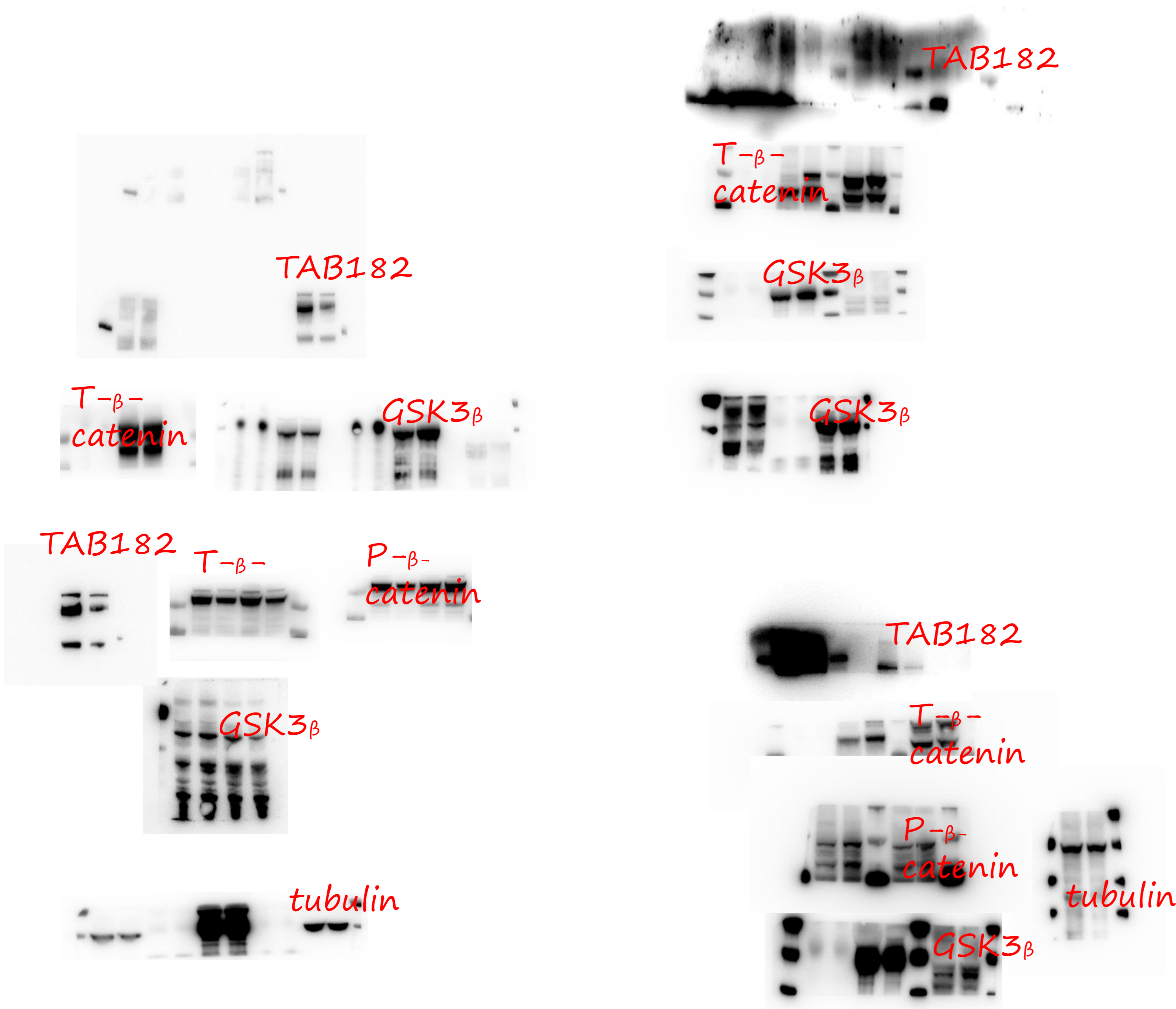

I

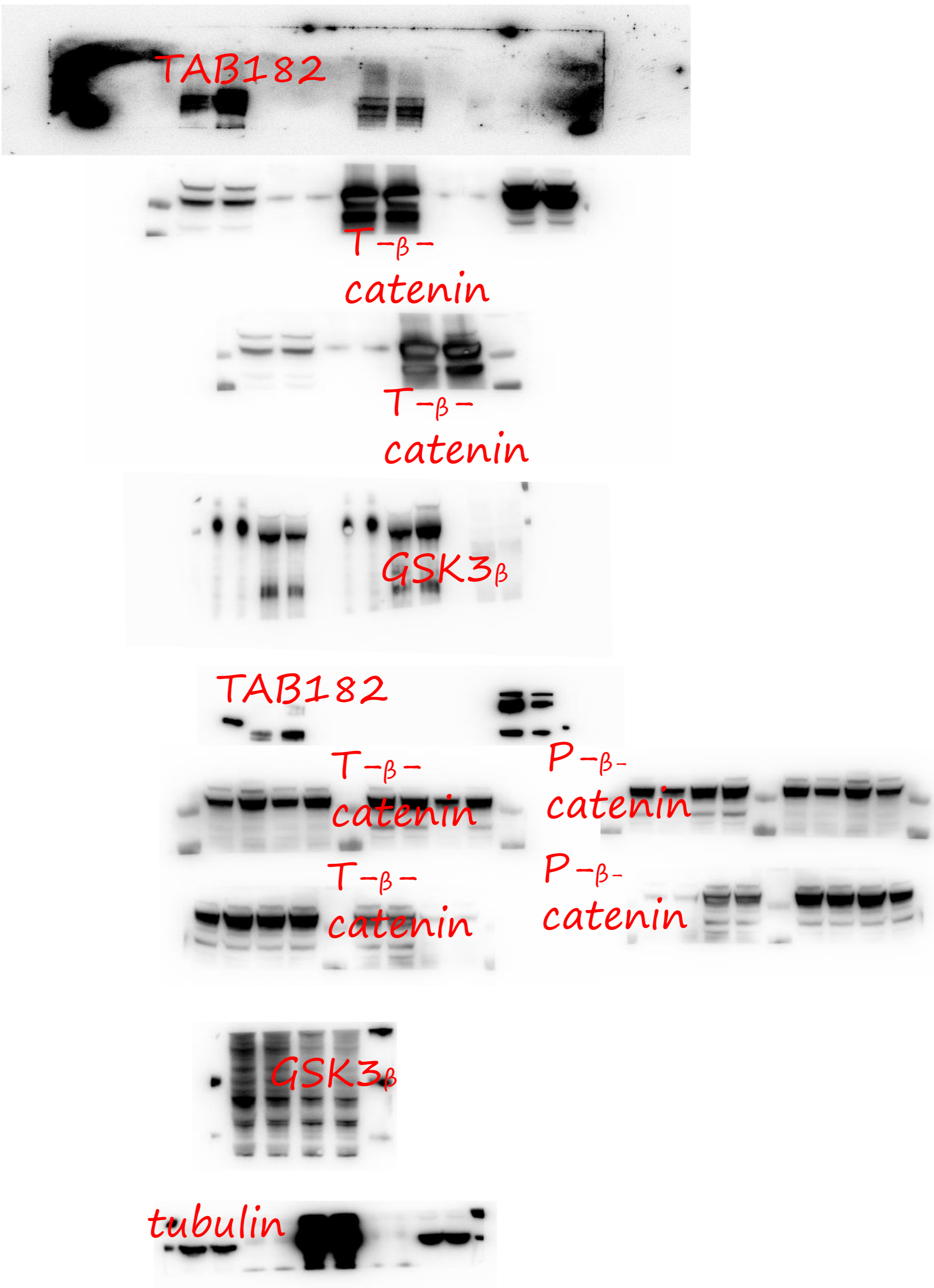

Figure 4

A

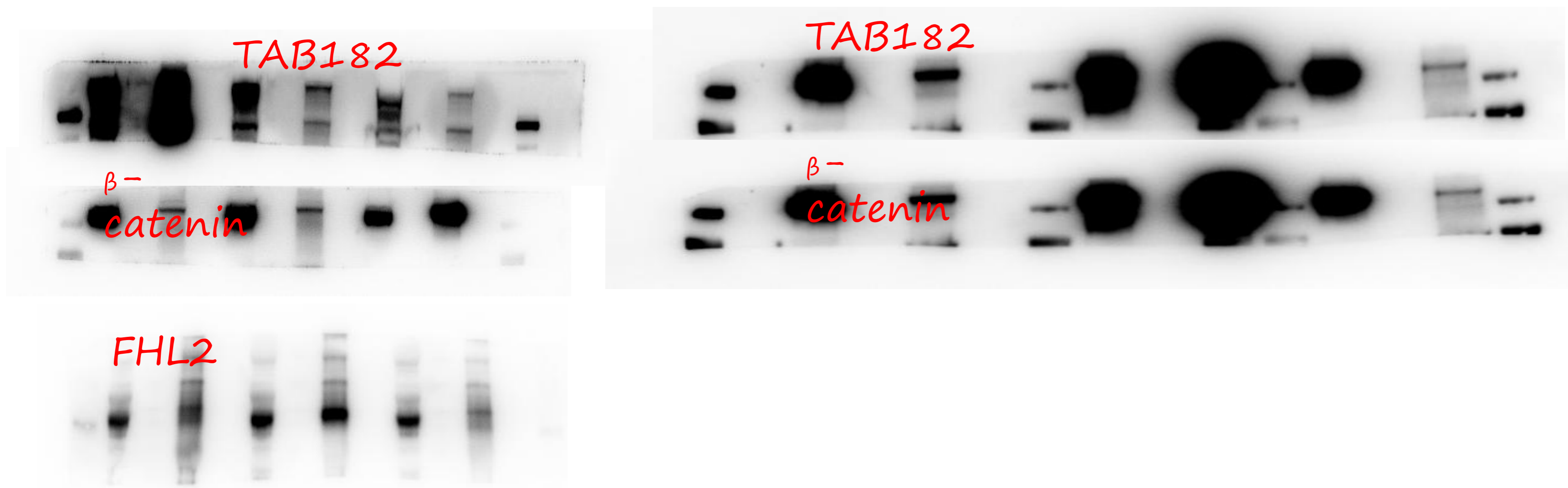

B

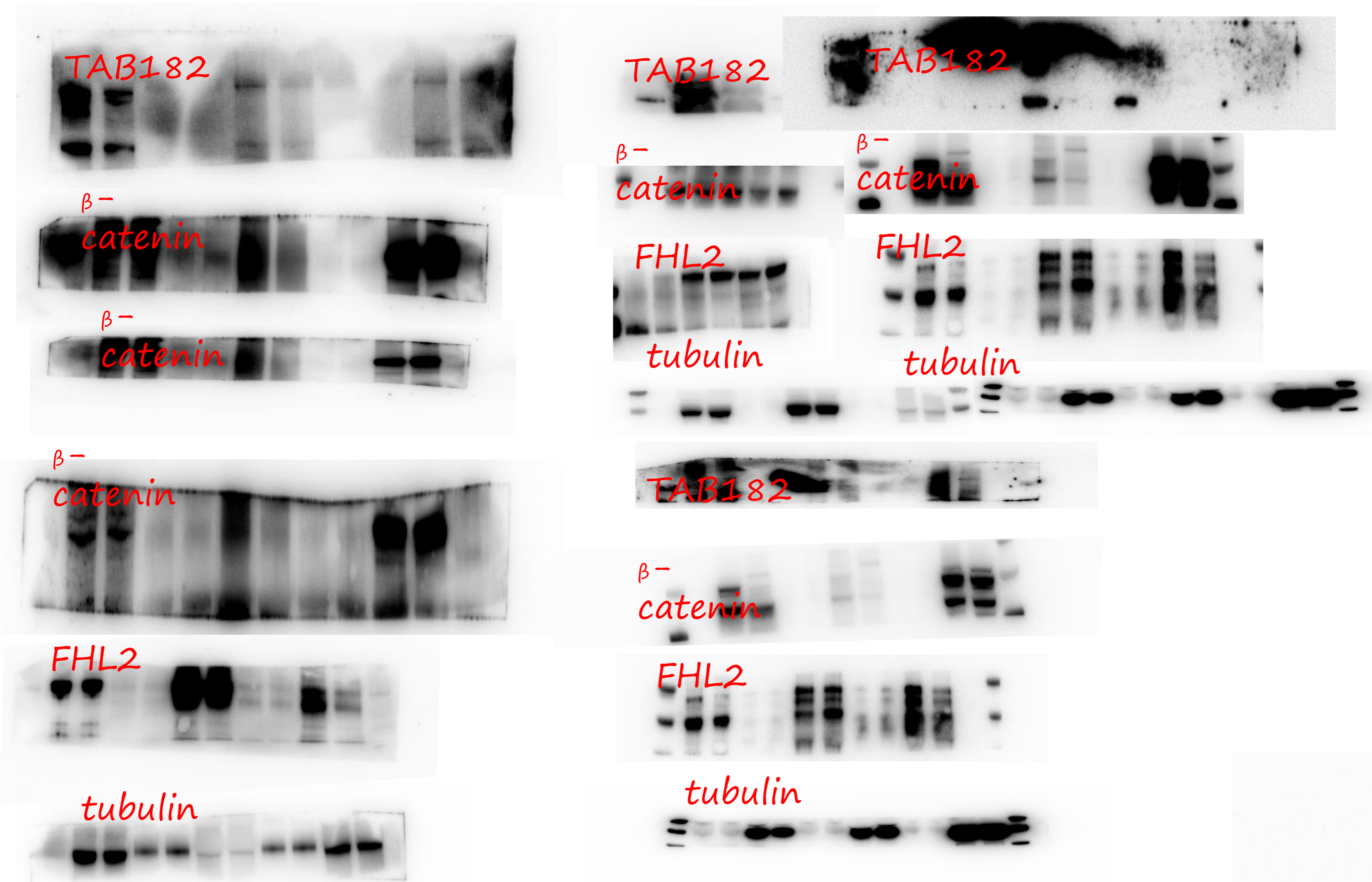

C

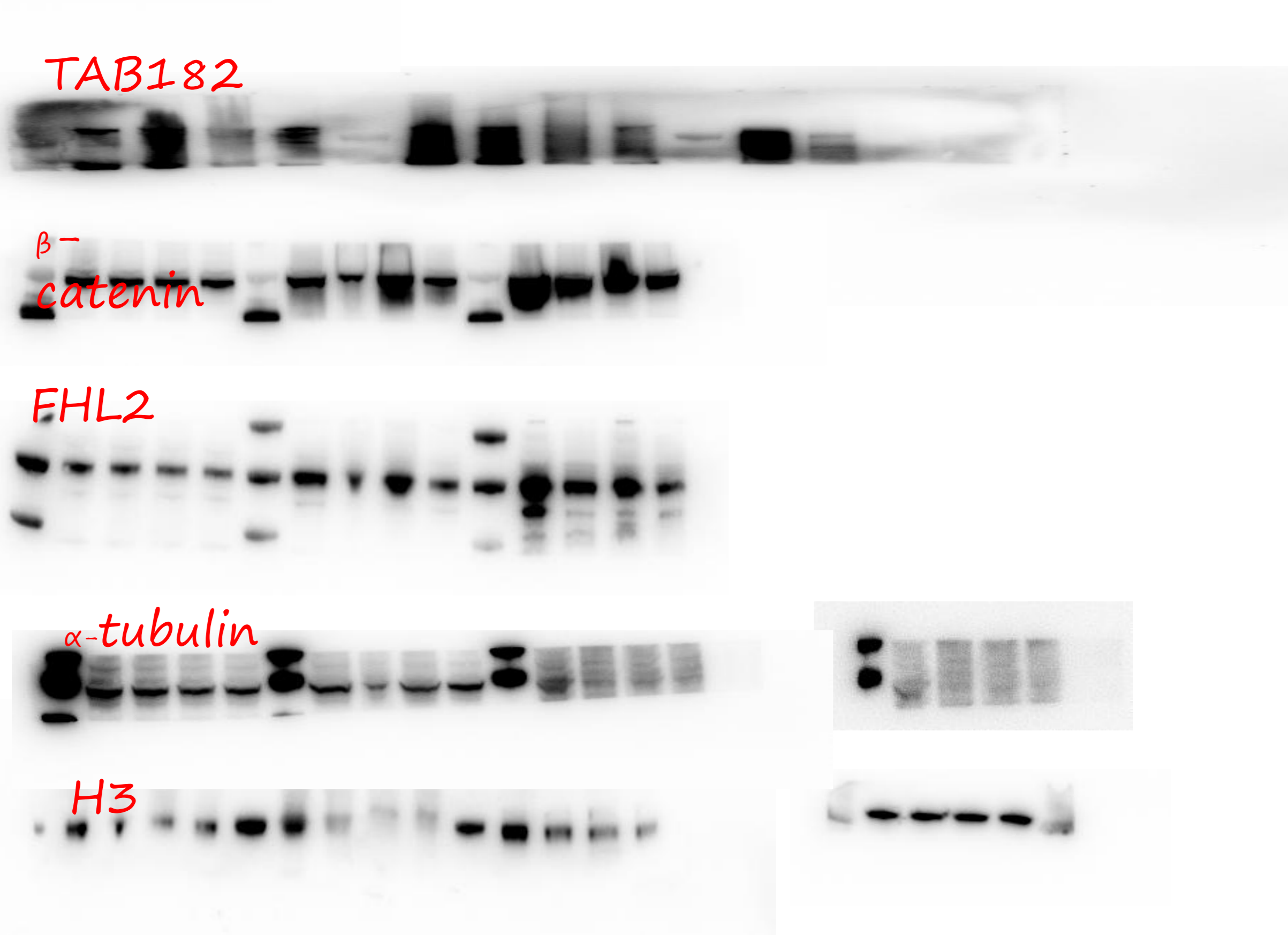

C

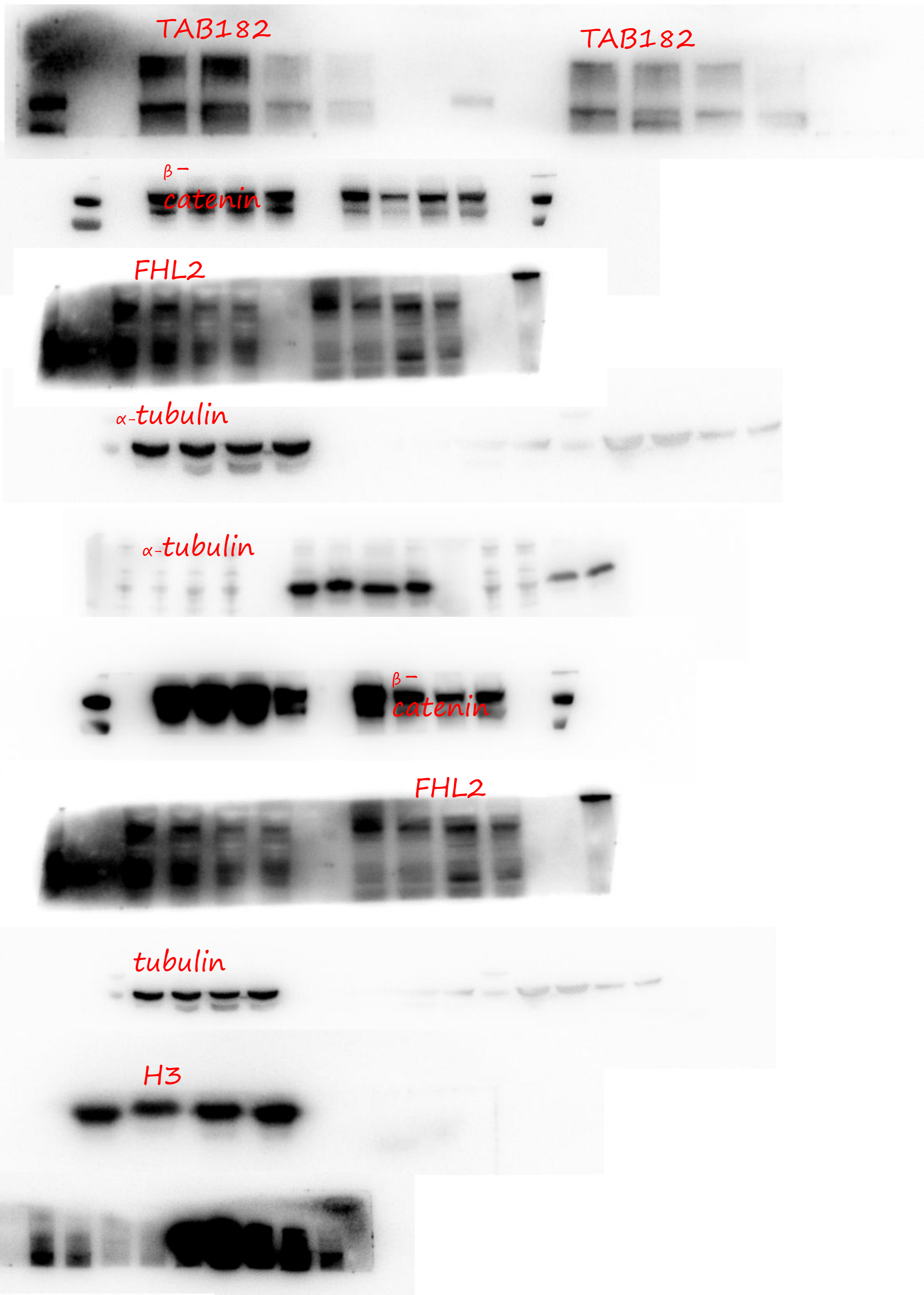

Figure 4

E

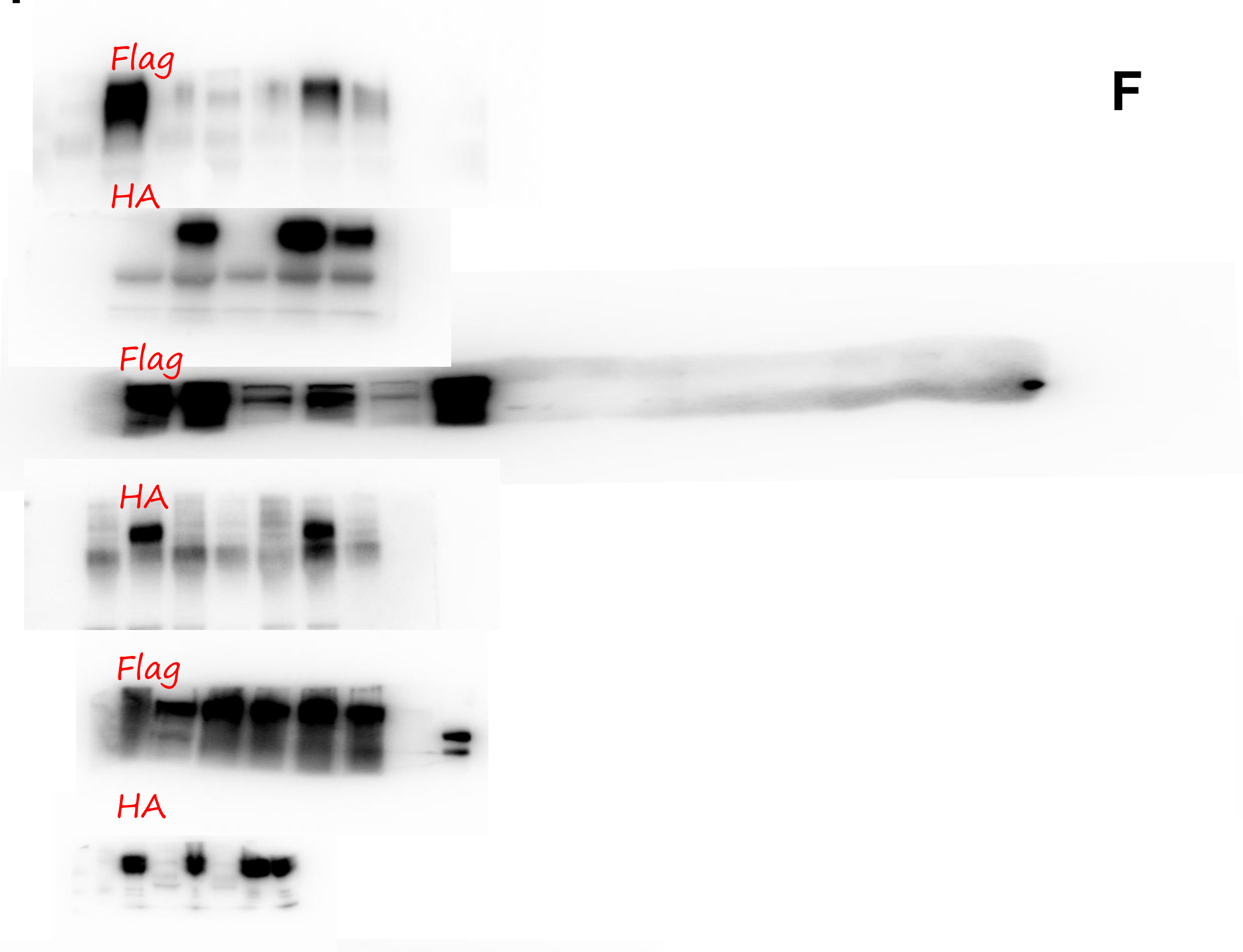

F

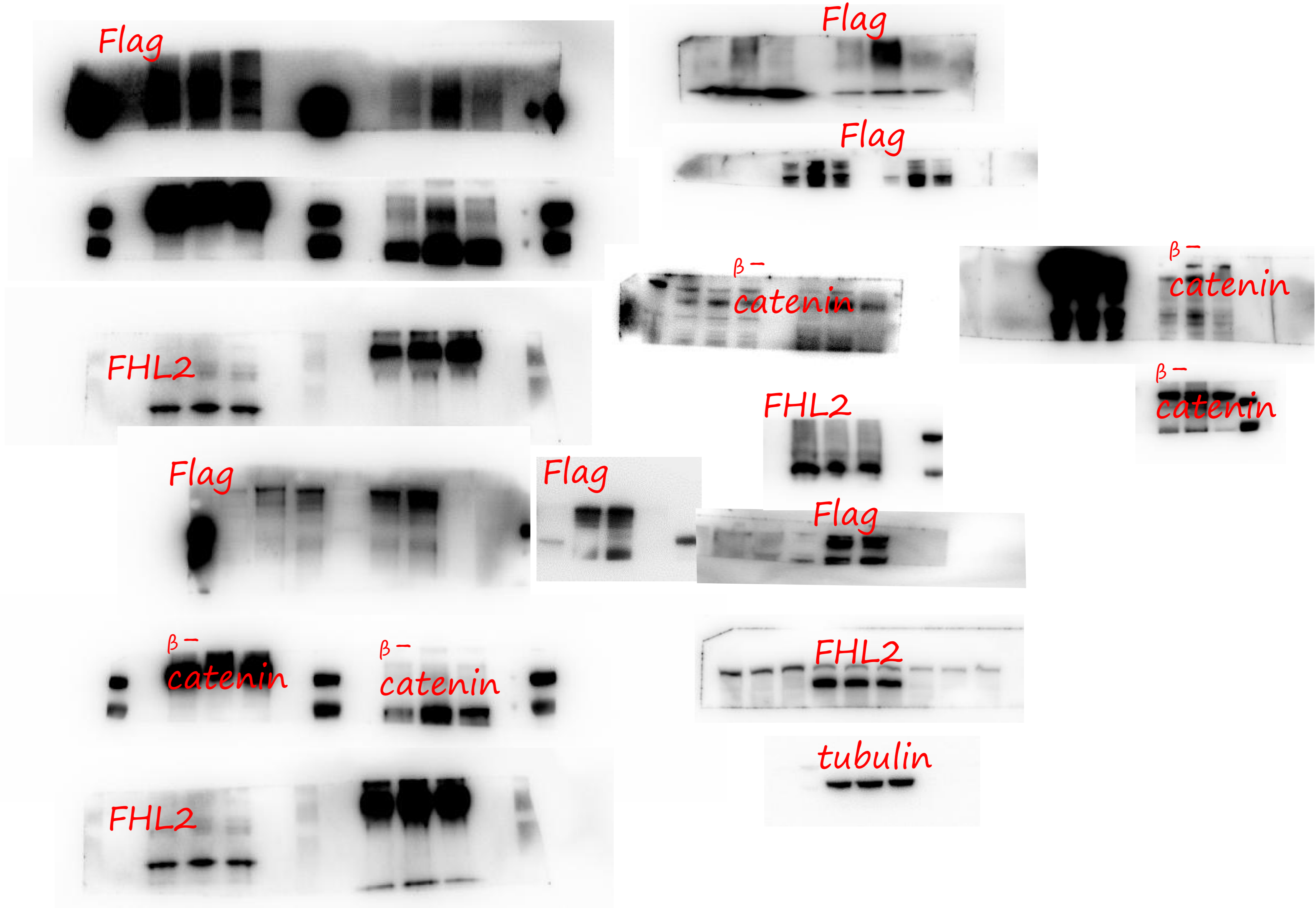

G

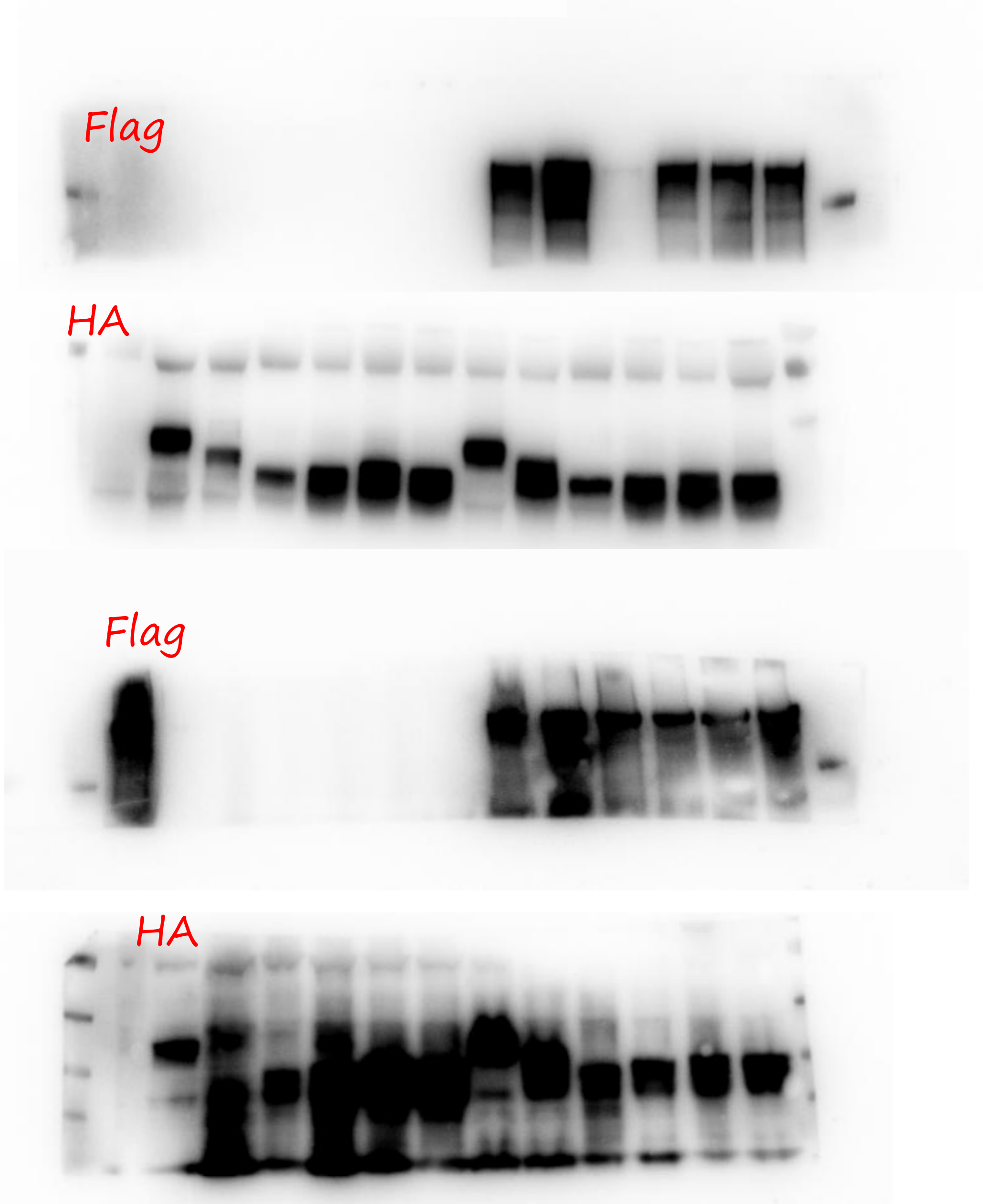

Figure5

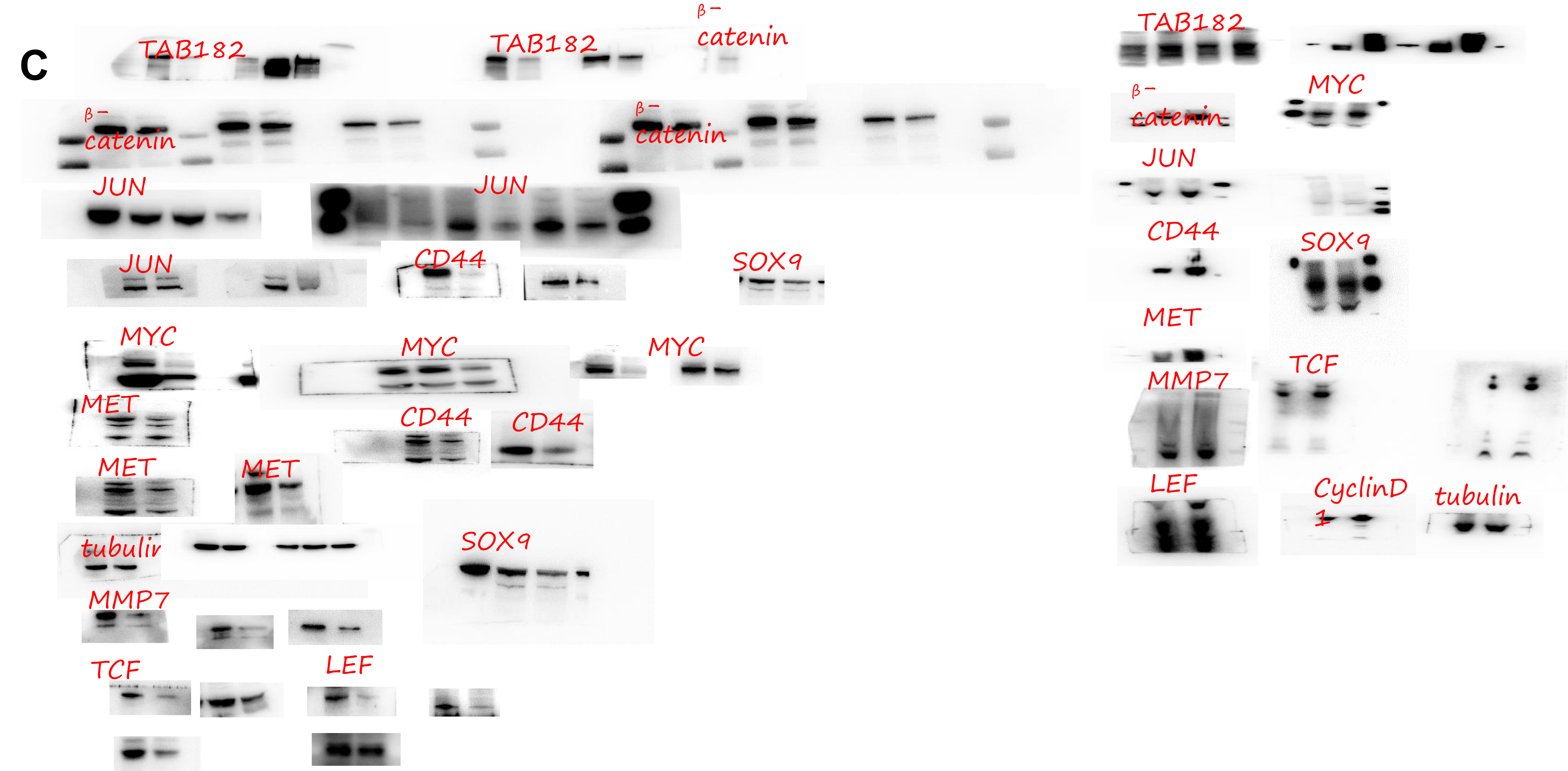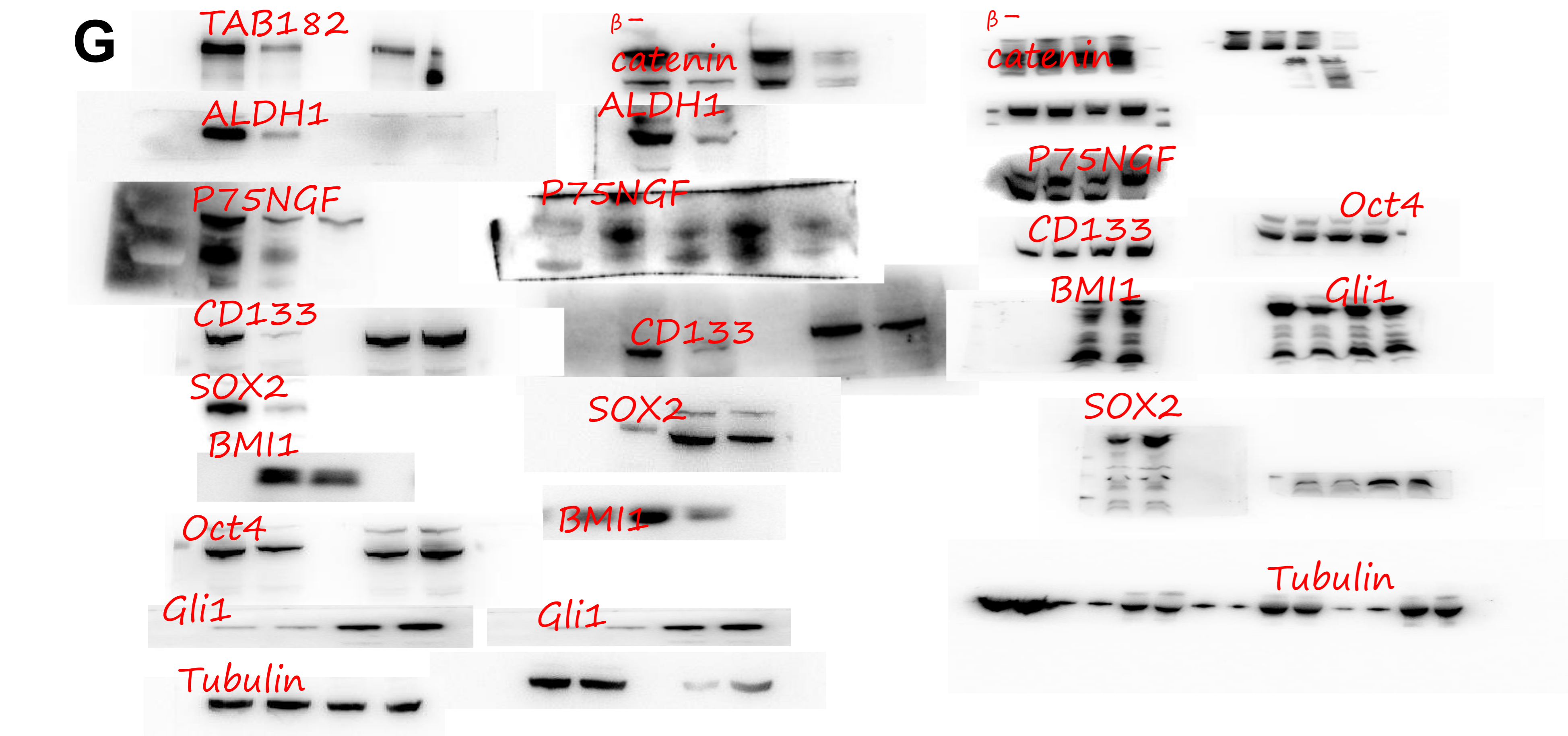

Figure6

E

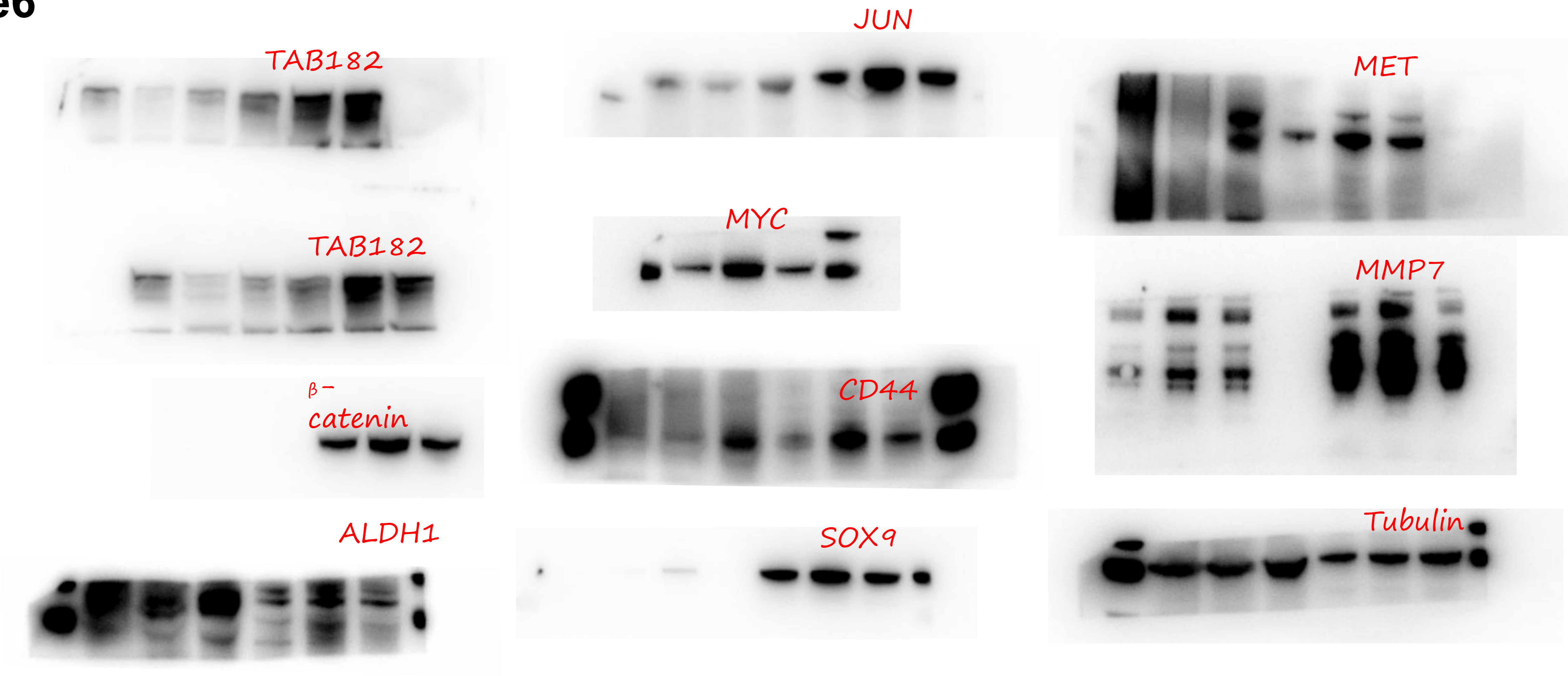

F

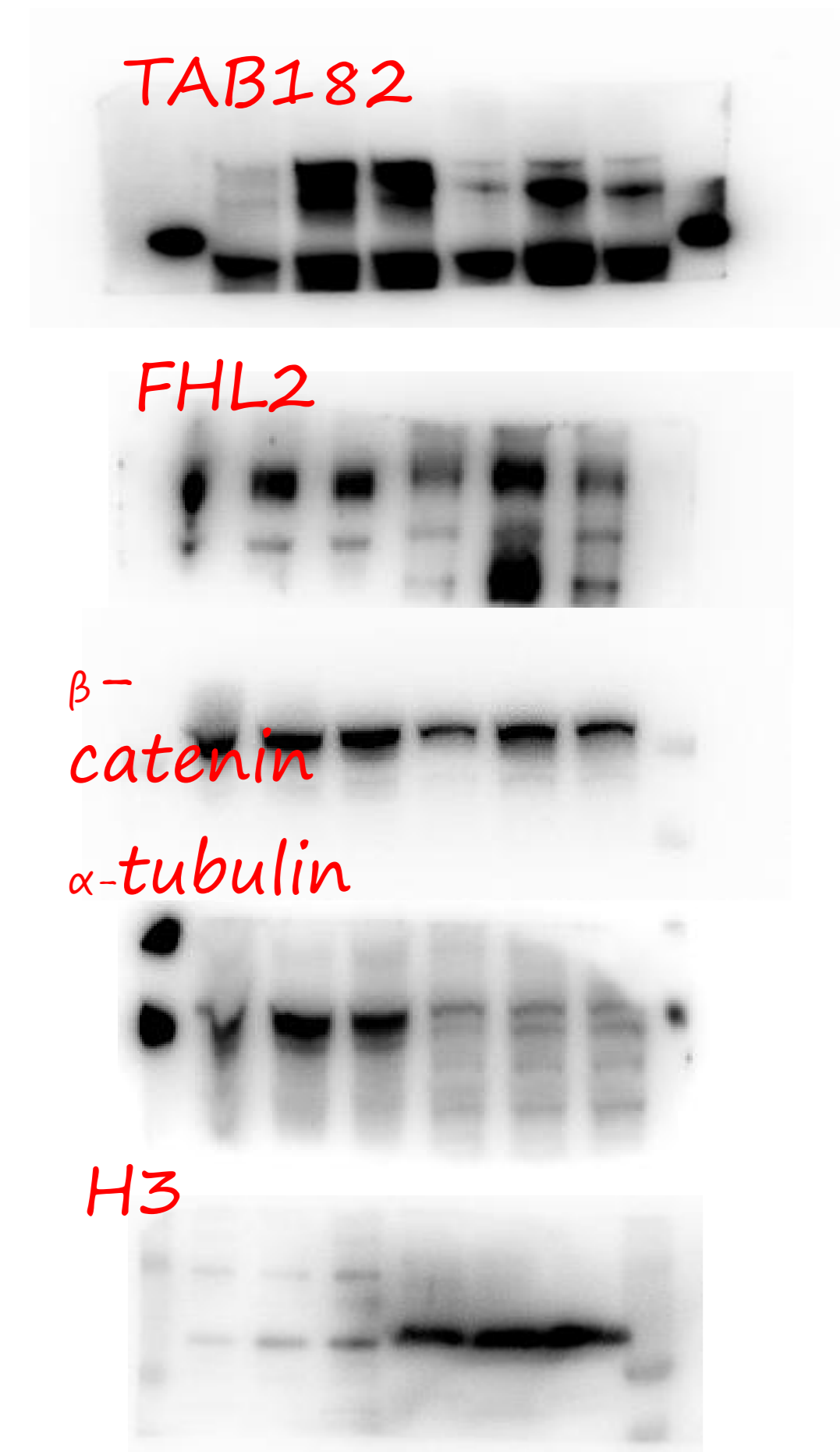

Figure7

B

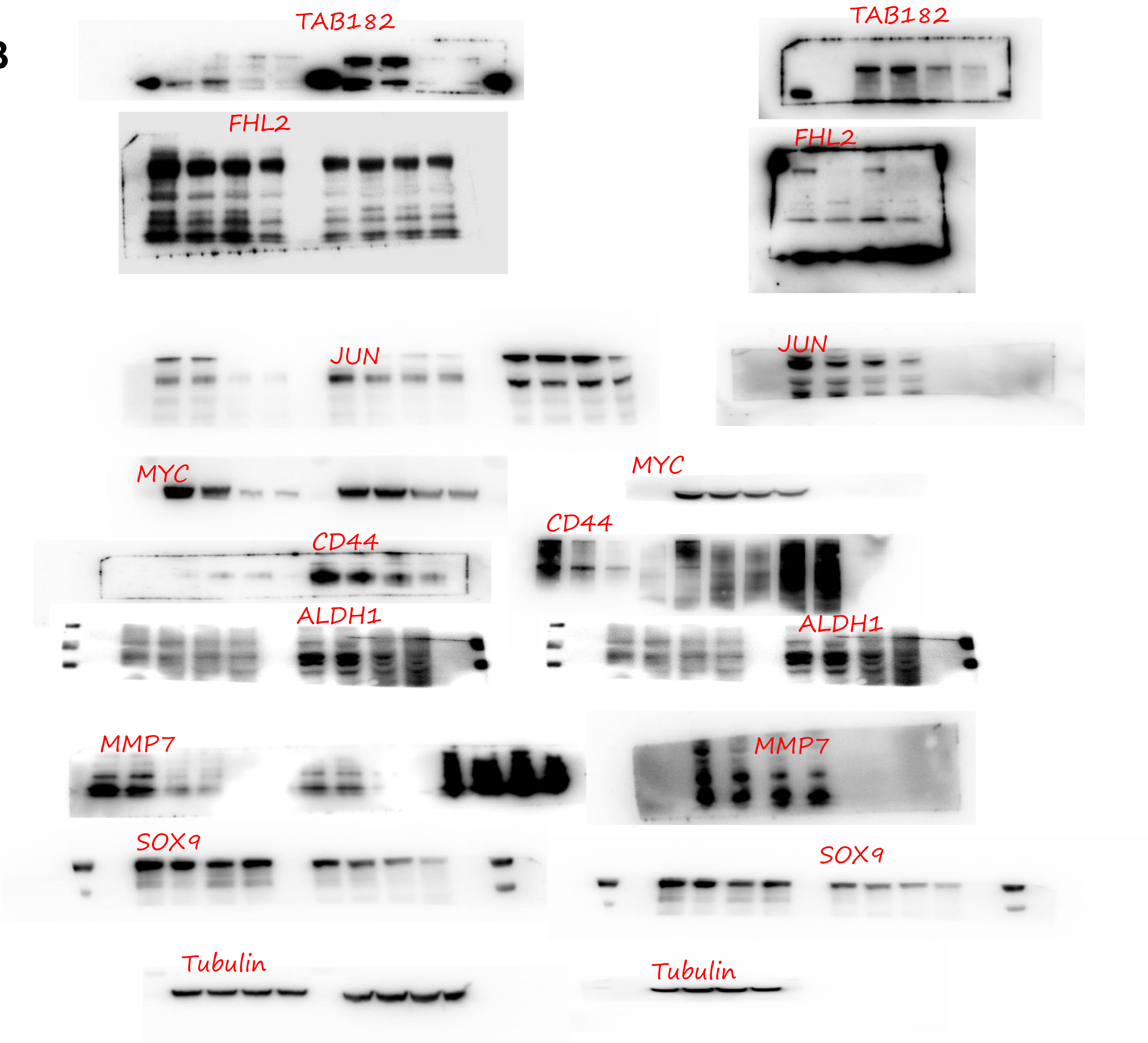

Supplementary Figure 1

B

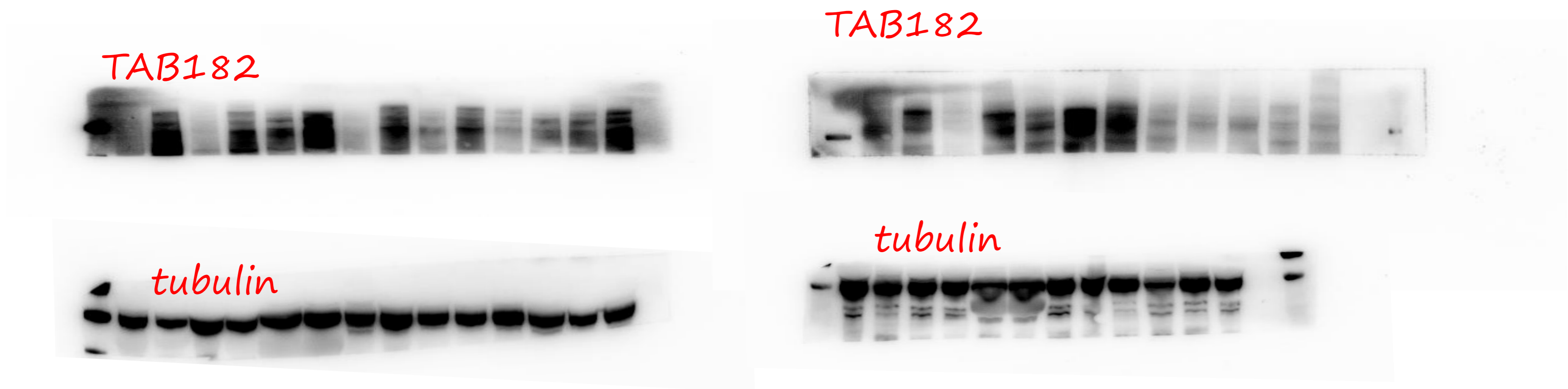

Supplement: Supplementary file 7 — Full Western Blots [file 41419_2022_5334_MOESM7_ESM.pdf]
